# Stony Coral Tissue Loss Disease Results in Persistent Microbial‐Level Disturbances on Coral Reef Ecosystems

**DOI:** 10.1111/1758-2229.70264

**Published:** 2025-12-21

**Authors:** Stephanie M. Rosales, J. Grace Klinges, Abigail S. Clark, Erinn M. Muller, Lindsay K. Huebner

**Affiliations:** ^1^ Cooperative Institute for Marine and Atmospheric Studies, University of Miami Miami Florida USA; ^2^ Atlantic Oceanographic and Meteorological Laboratory, National Oceanic and Atmospheric Administration Miami Florida USA; ^3^ Center for Global Discovery and Conservation Science, Arizona State University Hilo Hawaii USA; ^4^ Elizabeth Moore International Center for Coral Reef Research & Restoration, Mote Marine Laboratory Summerland Key Florida USA; ^5^ Scouting America, Sea Base, Brinton Environmental Center Summerland Key Florida USA; ^6^ Mote Marine Laboratory Sarasota Florida USA; ^7^ Fish and Wildlife Research Institute, Florida Fish and Wildlife Conservation Commission St. Petersburg Florida USA

**Keywords:** co‐occurrence, ecosystem disturbance, endemic, epidemic, Florida's Coral Reef, SCTLD, vulnerable

## Abstract

Stony coral tissue loss disease (SCTLD) has reduced coral diversity and homogenised benthic communities. Beyond coral loss, SCTLD may disrupt microbiome composition and function, affecting reef recovery. We examined microbiome changes of apparently healthy corals, water, and sediment at three patch reefs in the Lower Florida Keys during three SCTLD stages: before (vulnerable), during (epidemic), and after (endemic) the outbreak. SCTLD significantly altered microbial diversity and functional potential within apparently healthy corals and the surrounding reef environment. In corals, microbial alpha and beta diversity were highest at the vulnerable stage before declining by the endemic stage, indicating lingering impacts of SCTLD on microbial diversity. Network neighbour and betweenness analyses revealed a loss in connectivity in microbial communities in coral and sediments during the endemic stage. Microbial functional prediction indicated an increase in multidrug resistance and sulphur cycling genes in corals in the epidemic stage. Predicted nitrogen fixation genes were enriched in epidemic coral and seawater, and endemic coral and sediments. SCTLD‐associated taxa increased in apparently healthy corals, water and sediments during the epidemic stage, with some taxa persisting in the reef environment during the endemic stage. Thus, SCTLD likely has lasting taxonomic and functional microbial disruptions in coral reef ecosystems.

## Graphical abstract

Reef microbiomes before the arrival of stony coral tissue loss disease (vulnerable stage) during the outbreak (epidemic) and after (endemic). Microbial diversity, network metrics, and functional potential varied among apparently healthy corals and the surrounding water and sediments across the stages. These patterns indicate lasting microbial disruptions in coral reef ecosystems from this disease.

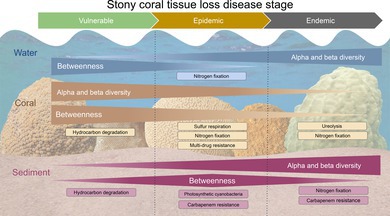

## 1 Introduction

Since its initial detection in September 2014 off the coast of Miami, Florida, stony coral tissue loss disease (SCTLD) has spread throughout Florida's Coral Reef and into the Caribbean (Alvarez‐Filip et al. 2019; Precht et al. 2016; Alvarez‐Filip et al. 2022). This disease has affected at least 22 species of scleractinian corals, significantly impacting coral reef communities (Swaminathan et al. 2024; Toth et al. 2023; Alvarez‐Filip et al. 2022). The wide host range of SCTLD has led to a decline in coral species richness and a notable decrease in mean colony size (Hayes et al. 2022). Further, the loss of SCTLD‐susceptible corals has increased the cover of weedier members of the benthic community, such as fire coral, macroalgae, and cyanobacteria (Swaminathan et al. 2024), correlating with increased ammonium (Becker et al. 2024). These changes have resulted in a more homogenous benthic community at both the taxonomic and functional levels (Alvarez‐Filip et al. 2022) and a decline in reef accretion potential (Toth et al. 2023), negatively impacting fish and other organisms that use coral reef habitats (Swaminathan et al. 2024).

In addition to changes in nutrient levels and benthic and fish communities, the composition of microbial communities is potentially altered during and after SCTLD outbreaks (Rosales et al. 2023, 2020; Clark et al. 2021). There are multiple microbial associates in SCTLD lesions, such as Rhodobacterales, Rhizobiales, Clostridia, and *Vibrio*, and while perhaps not the pathogen(s) responsible for SCTLD themselves, they are likely players in the microbial dynamics of the disease (Rosales et al. 2023; Heinz et al. 2024; Arriaga‐Piñón et al. 2024). In addition to being enriched in diseased corals on affected reefs, some SCTLD‐associated bacteria are detected at significant levels in healthy corals, sediments and seawater at the same sites. In contrast, during the same time point, healthy corals, seawater and sediments from reefs not yet exposed to SCTLD showed little to no presence of these taxa (Rosales et al. 2023, 2020; Clark et al. 2021). Consequently, similar to the changes in benthic and fish community composition, we hypothesize that SCTLD also threatens the structure and function of coral reef microbial communities.

The pace of SCTLD spread along Florida's Coral Reef had a relatively rapid expansion moving northward from Miami (Muller et al. 2020). However, it was more gradual and better monitored moving southward through the Upper, Middle and Lower Florida Keys and Dry Tortugas (Muller et al. 2020; Dobbelaere et al. 2020). This allowed researchers to document reef changes through three SCTLD stages: vulnerable, where SCTLD had not yet been reported; epidemic, where SCTLD was active and widespread; and endemic, where SCTLD had passed through, with few or no active lesions remaining (Dobbelaere et al. 2020). The contagious spread of SCTLD is indicative of a biotic pathogen transmitting through the water column (Aeby et al. 2019; Muller et al. 2020; Dobbelaere et al. 2020), likely associated with suspended sediments, other particles and biofilms originating from coastal development and/or shipping activities (Miller et al. 2016; Cunning 2019; Dobbelaere et al. 2020; Studivan et al. 2022; Evans et al. 2022). Accordingly, in the Florida Keys, distinct bacterial groups were identified in seawater and sediment at reefs across the stages of SCTLD (vulnerable, epidemic, and endemic) (Rosales et al. 2020; Clark et al. 2021), indicating that SCTLD has persistent effects on the reef microbiome. While these shifts may be less visible than changes in coral and fish communities, they likely have significant implications for overall reef ecosystem health.

Previous studies have contributed to our understanding of how SCTLD affects the microbiomes of coral reef environments. However, when comparing contemporaneous samples from reefs in different SCTLD stages, it has been challenging to distinguish the impacts of geographical factors from those of the disease itself. To better determine how SCTLD affects the reef microbiome and identify consistent microbial signatures reflective of SCTLD, we examined how the microbiomes of apparently healthy corals, seawater and sediment changed at three Lower Keys reefs, first sampled in June 2018 when in the vulnerable stage (Rosales et al. 2020; Clark et al. 2021), as they transitioned through the epidemic and then endemic stages. This study focused on healthy coral colonies to understand microbial changes that may be engendered by the presence of disease outside of causing coral tissue loss. Specifically, we (1) investigated microbiome diversity and co‐occurrence shifts in apparently healthy corals, seawater and sediment over time, as reefs progressed through SCTLD stages, (2) predicted functional changes in microbial communities in response to SCTLD and (3) assessed bacterial biomarkers associated with SCTLD from a meta‐analysis (Rosales et al. 2023) across samples from the three Lower Keys sites at different disease stages, and characterise their persistence in the environment.

## 2 Experimental Procedures

### 2.1 Benthic Habitat Relative to SCTLD Stages at Lower Keys Patch Reefs

The spread of the SCTLD spatial boundary through the Florida Keys was monitored by the collective efforts of agencies and organisations through frequent in situ reconnaissance of active SCTLD lesions on corals (‘Florida's Coral Reef Disease Outbreak Response’; FKNMS, n.d.). Sites ahead of this boundary, at which no corals had yet developed SCTLD lesions, were termed vulnerable to the spread of the disease. After SCTLD lesions appeared at a site and disease prevalence increased, the site was considered in the epidemic stage of the disease. This stage, characterised by disease prevalence of up to 55% of coral colonies in the Florida Keys, can last ~5–6 months at a site before incidence of new lesions slows (Sharp et al. 2020; Williams et al. 2021). Thereafter, the site is in the endemic stage of SCTLD.

To create an SCTLD stage‐based sampling design, we leveraged two existing SCTLD microbiome studies (Clark et al. 2021; Rosales et al. 2020) that combined examined the microbiomes of apparently healthy coral, seawater and sediment at three Lower Keys patch reef sites (Xesto Patch, Lindsay's Patch and Cliff Green) when the sites were in the vulnerable stage of SCTLD in May–June 2018. Based on the ongoing reconnaissance of the SCTLD boundary, the disease arrived at the area of these focal sites in the summer of 2019. To characterise the transition of these sites from the vulnerable stage into the epidemic and endemic stages, and to place our microbiome data into a reef community context, we referenced two large‐scale coral reef monitoring programs: the Coral Reef Evaluation and Monitoring Project (‘Coral Reef Evaluation and Monitoring Project (CREMP)’, n.d.), and Disturbance Response Monitoring (‘Disturbance Response Monitoring (DRM)’, n.d.). CREMP annually monitors fixed reef sites in a repeated measures design, allowing for precise assessments of community change through time. CREMP includes assessments of the percent cover of benthic groups and coral demographics and condition within four transects at each site. DRM surveys coral demographics and condition within two transects at random sites stratified across habitats during August to October. To examine SCTLD incidence at Lower Keys patch reefs, we used DRM data to generate counts of any coral colonies with SCTLD from 2014 to 2023. To examine the community change in response to SCTLD stage, CREMP data from 2014 to 2023 were analysed for percent cover of major benthic groups, and for the number of colonies and live tissue area (LTA) of focal coral species, focusing on the following Lower Keys patch reefs near our microbiome study sites: Red Dun Reef, West Washerwoman, Western Head and Cliff Green (which was also one of our sampling sites). Coral species data were examined for the species evaluated in this study, as well as for seven additional coral species groups known to be susceptible to SCTLD or common at the sites. Due to the potential of observers to misidentify the three species within each of the *Orbicella* and *Mycetophyllia* genera, these species were grouped by genus for examinations of coral colony counts and LTA.

### 2.2 Sample Collection and Processing

**FIGURE 1:**
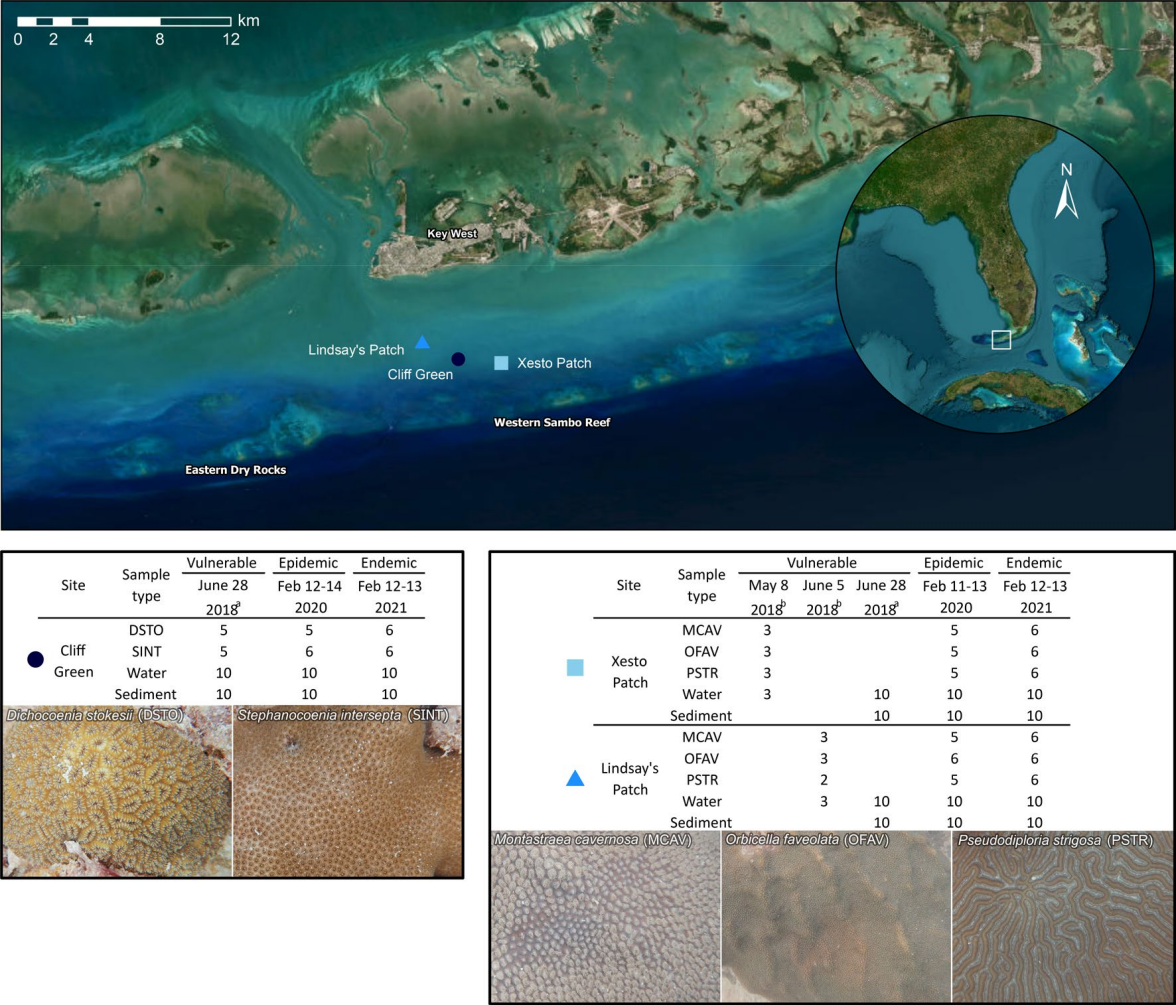
Three Lower Florida Keys patch reefs were sampled during the vulnerable, epidemic, and endemic stages of stony coral tissue loss disease (SCTLD). Summary of the sampling dates, the number of apparently healthy coral tissue, seawater, and sediment samples collected from each site. Coral species sampled are as follows: *Dichocoenia stokesii (DSTO), Montastraea cavernosa* (MCAV), *Orbicella faveolata* (OFAV), *Pseudodiploria strigosa* (PSTR), and *Stephanocoenia intersepta* (SINT). Vulnerable‐stage samples were from ^a^Rosales et al. (2020), and ^b^Clark et al. (2021), and epidemic and endemic‐stage samples were newly collected for this study.

We conducted a repeated site sampling design to assess apparently healthy coral, seawater, and sediment microbiome changes in response to SCTLD at the three Lower Keys patch reefs that were previously sampled when in the SCTLD‐vulnerable stage (Clark et al. 2021; Rosales et al. 2020): Xesto Patch, Lindsay's Patch, and Cliff Green (Figure 1 and Table S1). At each of Xesto and Lindsay's Patches, three samples of seawater and 2–3 samples of apparently healthy corals per species were collected in May–June 2018; the species sampled were *Colpophyllia natans, Montastraea cavernosa* (MCAV), *Orbicella faveolata* (OFAV), *Pseudodiploria strigosa* (PSTR), and *Siderastrea siderea* (Clark et al. 2021). At Cliff Green, five samples each of apparently healthy colonies of *Dichocoenia stokesii* (DSTO), *Diploria labyrinthiformis,* and *Stephanocoenia intersepta* (SINT) were collected in June 2018 (Rosales et al. 2020); 10 samples each of sediment and seawater were also collected at all three sites (this resulted in a total of 13 seawater samples from Xesto and Lindsay's Patches, at which seawater was sampled for both Clark et al. 2021 and Rosales et al. 2020; Figure 1 and Table S1).

In February 2020, after these sites had progressed to an epidemic stage of SCTLD, we resampled, taking 10 samples each of sediment and seawater (Figure 1). Based on colony availability and logistical reasons, we sampled 5–6 apparently healthy colonies per species of a subset of the previously sampled coral species per site: MCAV, OFAV, and PSTR at Xesto and Lindsay's Patches, and DSTO and SINT at Cliff Green. We followed this same sampling design in February 2021, when the reefs had progressed to an endemic stage of SCTLD. The focal coral species and sample set per site analysed in this study are presented in Figure 1.

Sample collections for the epidemic and endemic stages were conducted as previously described for the vulnerable stage collections (Rosales et al. 2020; Clark et al. 2021). In brief, seawater samples were collected with 1‐L Pyrex bottles, about 20 cm above the substratum near coral colonies, and sediment samples were scooped from the surface layer of the substratum, also near coral colonies, into 5‐mL tubes. During the epidemic stage, sediment and water samples were gathered near and over, respectively, coral colonies with SCTLD lesions. Apparently healthy colony tissue samples were collected by scraping 10‐mL blunt‐tip syringes across the coral surface and filling the syringe with the denuded coral tissue and mucus slurry. On the boat, coral slurry samples were transferred to 15‐mL plastic tubes. All samples were held in a dark cooler on wet ice (for 2–6 h, depending on sampling day) for transport to Mote Marine Laboratory in Summerland Key, Florida, where the coral tissue and sediment samples were immediately flash‐frozen in liquid nitrogen and placed in a −80°C freezer for later processing. The water samples remained on wet ice until they were filtered through 0.2 μm filters, a process that took ~5 h to complete. After a water sample was filtered, the filter was immediately transferred to a Whirl‐Pak, flash‐frozen, and placed in a −80°C freezer.

### 2.3 Laboratory Processing

The new samples collected for this study (*n* = 210) were DNA extracted using the DNeasy PowerSoil Kit (QIAGEN, Germantown, MD, USA) with modifications to the manufacturer's protocol as previously described (Clark et al. 2021; Rosales et al. 2020). A total of 200 μL of solution was discarded from each DNeasy PowerBead tube. The starting volume of coral tissue/mucus slurries was standardised. For the water filters, each was cut in half, and one half was added to a PowerBead tube. For sediment samples, 0.25 g of each sediment sample was used and added to a PowerBead tube. For all samples, phenol: chloroform: isoamyl alcohol was added during the lysis step. DNA quantity and quality were assessed utilising a NanoDrop spectrophotometer (Thermo Fisher Scientific, Waltham, MA, USA), and then stored at −80°C. DNA isolates were submitted for high‐throughput 16S rRNA amplification, library prep, and sequencing to Mr. DNA Lab (Shallowater, TX, USA). Amplification of the 16S rRNA gene variable region (V4) was achieved using PCR primers 515F (GTGCCAGCMGCCGCGGTAA; Parada et al. 2016) and 806R (GGACTACVSGGGTATCTAAT; Archaea 806R; Takai and Horikoshi 2000), 1 μL of DNA, and the HotStarTaq Plus Kit (QIAGEN, Germantown, MD, USA). Thermal cycling conditions consisted of an initial denaturation at 94°C for 3 min, followed by 30 cycles of denaturation at 94°C for 30 s, annealing at 53°C for 40 s, and extension at 72°C for 1 min, with a final elongation at 72°C for 5 min. PCR products were multiplexed using unique dual index primers. After amplification, PCR amplified products were checked in a 2% agarose gel, measured with a Qubit, and then pooled together in equal molar concentrations. Pooled samples were purified using Ampure XP beads (Beckman Coutler, CA, USA) and used to prepare the Illumina DNA library. The pooled samples were processed with the MiSeq Reagent Kit v3 and underwent paired‐end sequencing on a MiSeq platform on 2 lanes (Illumina, San Diego, CA, USA). Previously collected samples (*n* = 93; Clark et al. 2021; Rosales et al. 2020) were processed using the same protocols and sequenced on two and six sequencing lanes, respectively.

### 2.4 Bioinformatic Analysis

For 16S rRNA analysis, existing sequences (*n* = 93) were already demultiplexed (Rosales et al. 2020; Clark et al. 2021). The newly collected samples for this study were demultiplexed with the program sabre v1 with the paired option (Joshi 2011). Once demultiplexed, all datasets (a total of 303 samples) were imported into Qiime2‐amplicon‐2024.10 (Bolyen et al. 2019) and the plugin Cutadapt 2.10 (Martin 2011) was used to remove forward and reverse primers using default parameters. To assign amplicon sequencing variants (ASVs), Qiime dada2 denoise‐paired 1.30.0 (Callahan et al. 2016) was run separately on each sequencing run. Based on a drop below Q30, the reads were trimmed at 200 bp for both forward and reverse reads. ASVs were given taxonomic assignments with the program classify‐sklearn and the SILVA 138‐99 database (Quast et al. 2013). Once sequences had their taxonomic assignments, those classified as mitochondrial or chloroplast were removed, and only the Bacteria and Archaea domains were selected. ASVs were removed if they had less than a 10‐count frequency in at least 10 samples. The resulting files were then imported into R 4.4.1 for further processing.

### 2.5 Alpha Diversity Analysis

The Shannon diversity index was used to evaluate how microbial community alpha diversity shifted with the SCTLD stage: vulnerable, epidemic, and endemic. First, apparently healthy coral (*n* = 117), reef water (*n* = 96) and sediment (*n* = 90) samples were separated. Each cohort was filtered to remove ASVs that summed up to less than 1 count across all samples. The phyloseq 1.46.0 function ‘rarefy even depth’ (Quast et al. 2013; McMurdie and Holmes 2013) was used to normalise read counts in coral (4654 reads), water (46,353 reads), and sediment samples (14,294 reads) based on minimum read depth from each cohort (Schloss 2024). The phyloseq ‘estimate_richness’ function was then used to generate the Shannon diversity index for each sample. The diversity indices were checked for normality, and normally distributed data were then tested with mixed linear models using the package lme4 1.1.35.3 (Gałecki and Burzykowski 2013). To assess the effects of disease stage on Shannon diversity, we compared multiple linear mixed‐effects models testing fixed and random effects. Model selection was based on restricted maximum likelihood (REML) criteria, residual variance, and significance of fixed effects. For corals, disease stage and season were used as fixed effects, and reef sites and coral species were used as random intercepts. For water and sediment samples, the final model included disease stage and season as fixed effects, with reef site as a random effect. Season (i.e., dry vs. wet) was set as a fixed effect to account for seasonal changes affecting the response variable (i.e., disease stage) (Zuur et al. 2009). Pairwise comparisons were conducted with the package multcomp 1.4.25 (Liu 2010) and tested with a general linear hypothesis (function glht) using TukeyHSD. All models were visually inspected with qqnorm and fixed versus residual values plots.

### 2.6 Beta‐Diversity Analysis

Changes in microbial composition through the vulnerable, epidemic and endemic stages across the three reefs were assessed by evaluating beta‐diversity. The data were categorised by apparently healthy corals across all species combined, as well as by individual species (DSTO, MCAV, OFAV, PSTR, and SINT), water, and sediment. To focus on prevalent ASVs, in each group, ASVs that summed to < 5 counts in 10% of the samples were removed from each cohort. Individual filtered count tables were centred‐log‐ratio (clr) transformed for normalisation. The Vegan 2.6.4 (Dixon 2003) adonis2 function with a Euclidean distance was used to test if beta‐diversity was significantly different among stages, with season used as a random effect. Pairwise comparisons of disease stages were then tested with pairwise adonis 0.4.1. A PERMANOVA analysis (using adonis) was also used to test whether the community composition varied significantly based on disease stage, coral species, project (i.e., Rosales et al. (2020), Clark et al. (2021), or new samples collected for this study), reef, date, season, and their interactions. To test microbial community variability of dispersion on SCTLD stage, the vegan function vegdist was used on the clr matrices with a Euclidean distance, followed by the function betadisper. Pairwise comparisons were then performed using TukeyHSD.

### 2.7 Random Forest Analysis

To identify which ASVs were associated with vulnerable, epidemic, and endemic site stages, a random forest classification (RFC) was performed with the R package randomForestSRC 3.2.2 (Ishwaran and Kogalur 2007). The samples were split into coral, water, and sediment, and then filtered to remove counts with < 5 counts in 10% of each cohort. For corals, the dataset consisted of 117 samples, with classes divided into 27 vulnerable, 42 epidemic, and 48 endemic. For water samples, 96 samples were split into 36 vulnerable samples and 30 epidemic and endemic samples each. There was a total of 90 sediment samples, with 30 samples in each stage. Each random forest model consisted of 500 trees grown using sampling without replacement. Model performance was evaluated using out‐of‐bag (OOB) error estimates derived from data excluded during resampling. The Gini impurity criterion with random splitting and 10 random split points per node guided tree construction. The importance of each ASV was computed using a permutation test. For each disease stage, the top 10 ASVs with the highest importance values were plotted for coral, water, and sediment.

### 2.8 Network Analysis

Random Forest outputs were also used to build networks to identify microbiome changes through time. ASVs that did not contribute to the overall model (i.e., importance = 0 or less) were removed. Subsequently, for coral, water, and sediment, ASVs were further separated into three groups: vulnerable, epidemic, and endemic (which had their respective importance values assigned). ASVs with zero or negative values within each group (i.e., importance within each group) were further filtered out. The remaining ASVs were used to build nine networks (one for each group type for each site stage) using the Stability Approach to Regularisation Selection (StARS) model in conjunction with Meinshausen–Bühlmann's neighbourhood selection method (Kurtz et al. 2015). For StARS, 100 subsamples were used. Node centrality was assessed using two metrics: ‘centrality_degree’ (‘Neighbours’, which measures the number of adjacent edges or connections) and ‘centrality_edge_betweenness’ (‘Betweenness’, which counts the number of shortest paths that pass through an edge and represents a bridge spanning role) (Brandes 2001; Madduri et al. 2009). The diameter of the networks (the maximum length of the shortest path between any two connected nodes within the network) was also measured to understand the closeness of the community. To identify significant differences in disease stages among each sample type in each network metric, the Wilcoxon Rank Sum Test was used with multiple comparisons corrected with the Bonferroni method (package rstatix 0.7.2). The package ‘influenceR’ 0.1.0 was used to identify the top five ASVs in each network designated as ‘key players’ (Borgatti 2006). These key players were then extracted and visualised through disease stages.

### 2.9 Functional Prediction Analysis

The predicted functional changes in microbial communities associated with SCTLD were assessed using two methods: PICRUSt2 (Douglas et al. 2020) and FAPROTAX (Louca et al. 2016). For PICRUSt2, data were categorised into coral, water, and sediment groups and filtered according to the procedures outlined for beta‐diversity. The picrust2_pipeline.py was then executed on each filtered dataset using default parameters. For FAPROTAX, the program microeco (Louca et al. 2016; Liu et al. 2020) was used by first converting the phyloseq objects into microeco objects; then the function cal_spe_func was used with the FAPROTAX database to assign function and their weighted abundance. Following both analyses, KEGG (Kanehisa and Goto 2000) and FAPROTAX matrices were transformed. After trials with different transformations, a log10 was selected to mitigate data skew. The transformed datasets were subsequently tested for differential abundance between disease stage and season as fixed effects with Maaslin2 (Caspi et al. 2019; Mallick et al. 2021) using linear mixed models. Coral species and reef sites were used as random effects for coral samples, and water and sediment samples; reefs were used as a random effect.

### 2.10 Identification of Known SCTLD‐Associated Bacterial Groups

To identify if bacterial groups found enriched in SCTLD‐diseased samples from our meta‐analysis study (Rosales et al. 2023) were present in these samples, we selected the 59 ASV sequences that were significantly differentially abundant in the previous analysis. These sequences were used to create a BLAST ‘SCTLD pathogen database’. The ASVs from the present study were then aligned with BLAST (Altschul et al. 1990) to the ‘SCTLD pathogen database’ using a 100% identity, −qcov_hsp_perc = 40 (given the difference in length) and an *e*‐value of 1 × 10^−5^. The sequences with similarity to the database were then selected for relative abundance analysis. The subset taxa were also analysed with the phyloseq ‘estimate_richness’ function to assess the number of SCTLD pathogen signatures (i.e., richness) observed in each sample. Significant differences in richness between disease stages within each sample type across reefs were determined using the Wilcoxon Rank Sum Test, with multiple comparisons corrected using the Bonferroni method.

## 3 Results

### 3.1 The Benthic Habitat Changes With SCTLD Stages at Lower Keys Patch Reefs

Our examination of Lower Keys patch reef coral data from the DRM program revealed the general alignments of our microbiome study sites from the vulnerable stage of SCTLD to the epidemic and then endemic stages. No colonies in this regional habitat exhibited SCTLD lesions in late summer (August–October) of 2018, but in 2019, the number of colonies with SCTLD lesions increased across the three late‐summer months (Figure S1a). This verifies the reports of the arrival of SCTLD to sites in this regional habitat in the summer of 2019 and that the transition into the epidemic stage, characterised by high disease prevalence, occurred in late 2019. Because the epidemic stage can last up to ~5–6 months in the Florida Keys (Williams et al. 2021; Sharp et al. 2020), our February 2020 sampling was within the timeframe of the epidemic stage. Qualitatively, numerous colonies across multiple species had visibly progressing SCTLD lesions at the time of sampling. The incidence of SCTLD lesions in the late summer of 2020 declined from that seen in 2019, indicating a transition to the endemic stage by our February 2021 sampling (Figure S1a).

The CREMP data, which were gathered in the summer months, revealed several changes in the benthic community in response to SCTLD at the four examined Lower Keys patch reefs (Figure S1b). Bare substrate cover increased gradually over time from 12% in 2018 and, following the SCTLD epidemic stage, substantially increased to over 15% in the epidemic stage in 2020 before declining in the endemic stage in 2021, but was still higher than before the outbreak. Stony coral cover was mostly stable at around 5% from 2014 to 2019, but from 2019 to 2020 and continuing through to 2021, coral cover declined to 4%. Macroalgae and cyanobacteria showed peaks in 2018 and 2015, respectively, and continuously declined through 2021. An examination of the coral species‐specific effects of SCTLD demonstrated more striking effects of the disease on the reef community. Coral counts for the focal species of this study declined from 965 individuals in 2018 to 855 in 2020 and 807 in 2021 (Figure S1c), with live tissue area (LTA) following a similar downward trend (Figure S1d). Other coral species affected by SCTLD also showed consistent decreases in coral counts and total LTA (Figure S1e,f). For example, three species considered highly susceptible to SCTLD, *Diploria labyrinthiformis* , *Eusmilia fastigiata,* and *Meandrina meandrites* , were not detected at all during either the epidemic or endemic phases compared with the vulnerable phase. Another highly susceptible species, *Colpophyllia natans* , exhibited a particularly sharp decline in LTA and abundance, with counts dropping from 64 in 2018 to just 13 in 2020 and 11 in 2021. *Siderastrea siderea* also showed declines, despite being the most highly abundant species and only intermediately susceptible to SCTLD. Finally, as a contrast, *Porites astreoides* , which is relatively abundant and has low susceptibility to SCTLD in Florida (Papke et al. 2024), showed slight increases in abundance and LTA during the epidemic and endemic stages of SCTLD.

### 3.2 Alpha Diversity Decreased in Healthy Corals Located in the Endemic Stage Compared With the Vulnerable and Epidemic Stages

**FIGURE 2:**
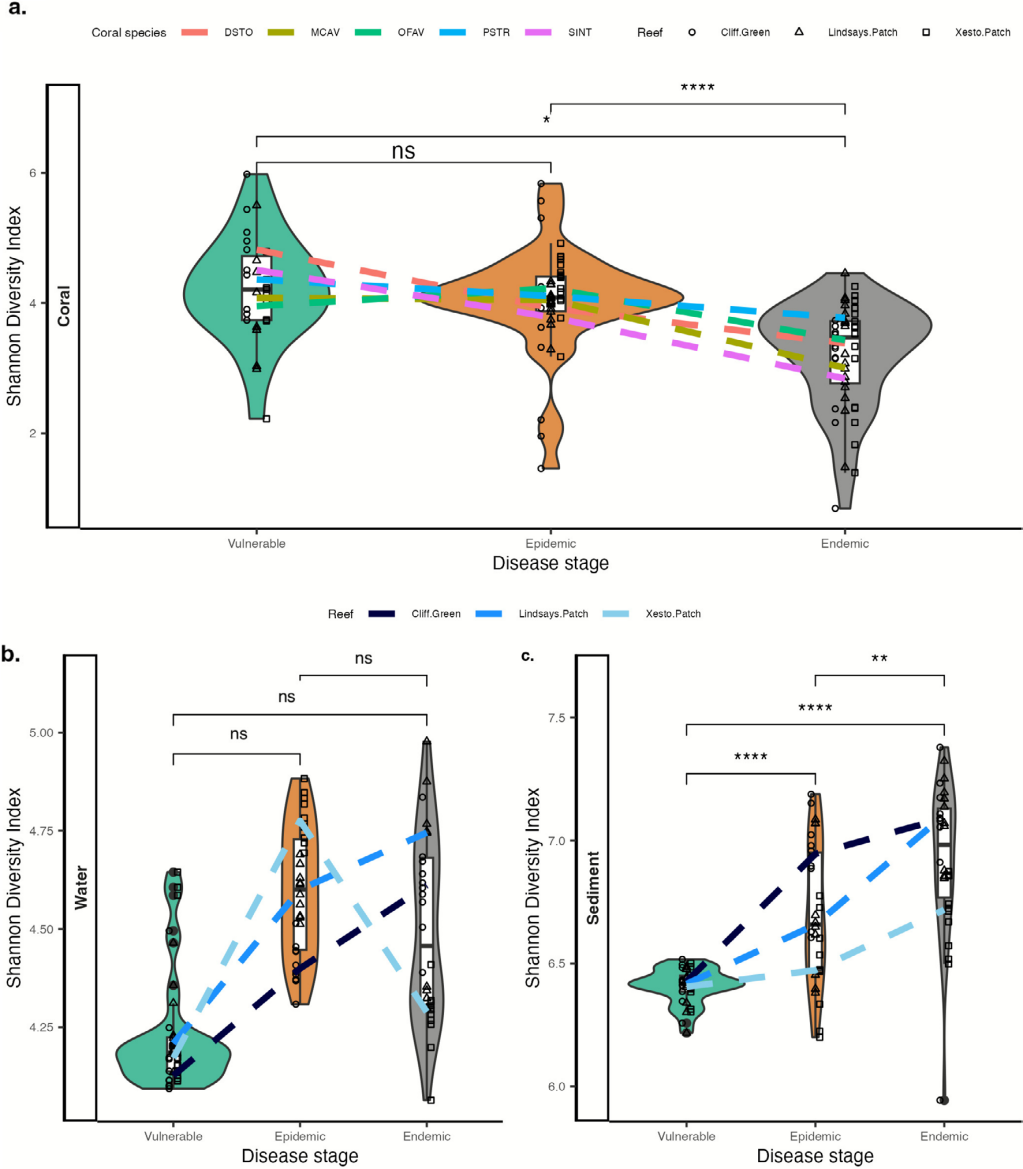
Alpha (Shannon) diversity across the three SCTLD stages for (a) coral, (b) water and (c) sediment is depicted. In panel (a), box plots show the combined data for all coral species, while the lines indicate the results for individual coral species: *Dichocoenia stokesii* (DSTO), *Montastraea cavernosa* (MCAV), *Orbicella faveolata* (OFAV), *Pseudodiploria strigosa* (PSTR) and *Stephanocoenia intersepta* (SINT). For panels (b) and (c), the box plots represent the combined samples, with lines showing results for each reef. In the box plots, the upper lines represent the 75th percentile, the lower lines represent the 25th percentile and the mid‐line indicates the median; the ‘whiskers’ extend to data points within 1.5 times the interquartile range from the maximum (upper) and minimum (lower) values. Each point represents a sample, with the point symbol indicating the reef from which the sample was collected; the shape of the violin plots indicates the distribution of the samples by Shannon diversity. Asterisks indicate significance in comparisons between disease stages; ‘ns’ indicates no significance.

We examined changes in microbial alpha diversity through SCTLD‐vulnerable, epidemic, and endemic stages across three reefs in the Lower Florida Keys. Corals in the epidemic stage did not differ significantly from those in the vulnerable stage in terms of diversity (epidemic–vulnerable = 0.13, SE = 0.30, *z* = 0.46, *p* = 0.89). However, corals in the endemic stage exhibited lower diversity compared with those in both the vulnerable (endemic–vulnerable = −0.71, SE = 0.29, *z* = −2.4, *p* = 0.0364) and epidemic stages (endemic–epidemic = −0.84, SE = 0.17, *z* = −5.0, *p* < 1e−04; Table S2 and Figure 2a).

### 3.3 Alpha Diversity Significantly Increased From Vulnerable to Epidemic and Endemic Stages in Sediments, With a Similar Trend in Water Samples

In water samples, although samples from the epidemic and endemic stages increased in alpha diversity compared with those from the vulnerable stage, there was no statistical difference between any pairwise comparisons (Table S2 and Figure 2b). For sediment samples, alpha diversity was higher during the epidemic (epidemic–vulnerable = 0.30, SE = 0.06, *z* = 5.3, *p* < 1e−04) and endemic stages (endemic–vulnerable = 0.52, SE = 0.05, *z* = 9.3, *p* < 1e−04) compared with the vulnerable stage. Differences were also found between the epidemic and endemic stages, with higher diversity in the endemic stage (endemic–epidemic = 0.22, SE = 0.06, *z* = 4.0, *p* = 0.0002; Table S2 and Figure 2c).

### 3.4 Within‐Group Variability Was Significantly Lower in Healthy Corals at the Endemic Stage, and Between Disease Stages There Were Discrete Clusters

**FIGURE 3:**
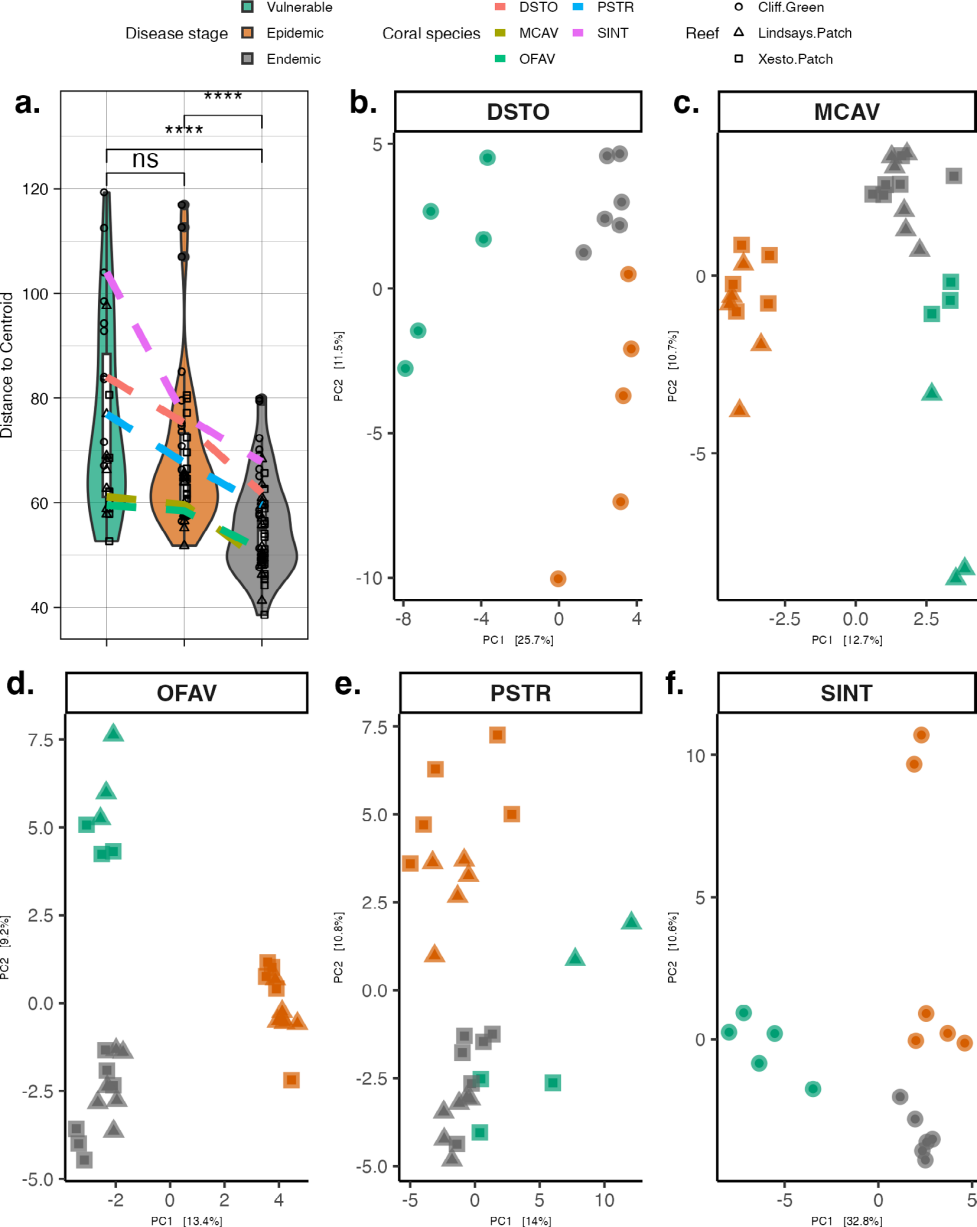
Beta‐diversity changes in coral microbiomes across SCTLD stages. (a) Community composition variability (i.e., dispersion) of all coral species and beta‐diversity community composition shifts of coral species using a Euclidean distance (b) *Dichocoenia stokesii* (DSTO), (c) *Montastraea cavernosa* (MCAV), (d) *Orbicella faveolata* (OFAV), (e) *Pseudodiploria strigosa* (PSTR), and (f) *Stephanocoenia intersepta* (SINT). In panel (a), the upper lines in the box plots represent the 75th percentile, the lower lines represent the 25th percentile, and the mid‐line indicates the median; the ‘whiskers’ extend to data points within 1.5 times the interquartile range from the maximum (upper) and minimum (lower) values. Each point represents a sample, with the point symbol indicating the reef from which the sample was collected; the shape of the violin plots indicates the distribution of the samples by beta‐diversity. Asterisks indicate significance in comparisons between disease stages; ‘ns’ indicates no significance.

When samples were combined across coral species, microbial community dispersion (within‐group community variability) was significantly higher during the vulnerable and epidemic stages of SCTLD compared with the endemic stage (*p*adj < 0.0001; Table S3 and Figure 3a). Individually, each coral species had a downward trend in dispersion from the vulnerable stage progressing to the endemic stage (Figure 3a); however, significant differences among stages varied per species (Table S3). Significant differences were found in the vulnerable stage to the epidemic (OFAV, *p*adj = 0.04) and the endemic stage (PSTR, *p*adj = 0.04). In the epidemic versus endemic comparisons, significant differences were found in three of the five corals: MCAV (*p*adj = 0.01), OFAV (*p*adj = 0.03), and PSTR (*p*adj = 0.02; Table S3). The microbial composition of each coral was also evaluated using a PERMANOVA, which indicated significant differences in community composition by disease stage in each coral species (DSTO, MCAV, OFAV, PSTR, SINT; Table S4 and Figure 3b–f). A principal component analysis of each species shows how the microbiomes of each species uniquely shifted among the three disease stages (Figure 3b–f).

### 3.5 Within‐Group Variability Was Lowest During the Vulnerable Stage in Sediment and Water Samples, and Both Had Significant Clustering Among Disease Stages

**FIGURE 4:**
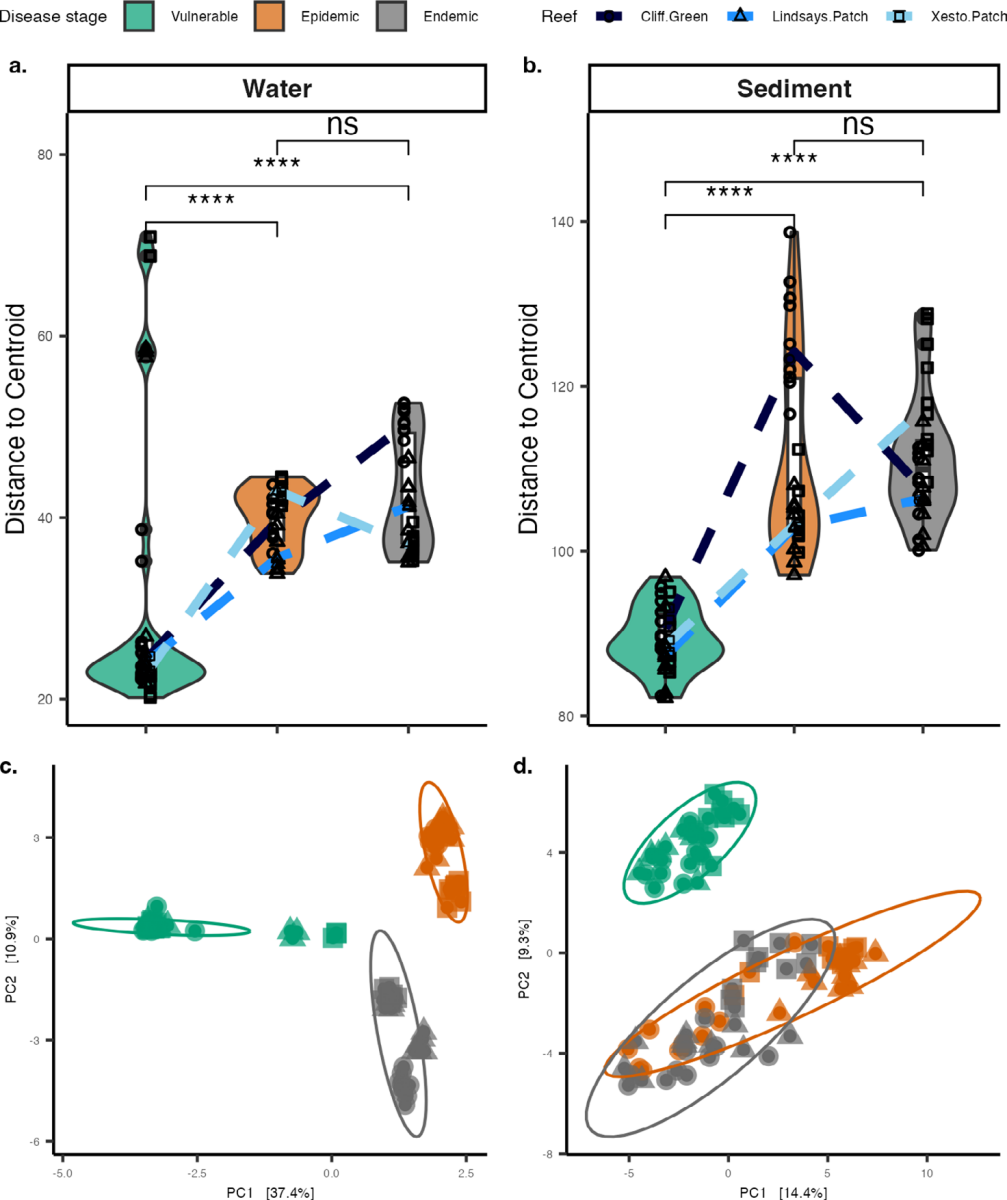
Beta‐diversity changes in coral reef water and sediment across SCTLD stages. Community composition variability (i.e., dispersion) of (a) water and (b) sediment. Beta‐diversity shifts using a Euclidean distance are shown for (c) water and (d) sediment. In panels (a) and (b), the upper lines in the box plots represent the 75th percentile, the lower lines represent the 25th percentile, and the mid‐line indicates the median; the ‘whiskers’ extend to data points within 1.5 times the interquartile range from the maximum (upper) and minimum (lower) values. Each point represents a sample, with the point symbol indicating the reef from which the sample was taken; the shape of the violin plots indicates the distribution of the samples by beta‐diversity. Asterisks indicate significance in comparisons between disease stages; ‘ns’ indicates no significance.

Both water and sediment samples exhibited similar dispersion patterns, showing increased within‐group microbial variability in the epidemic and endemic stages compared with the vulnerable stage. However, differences between the epidemic and endemic stages showed some reefs with increased variability and others decreased (Table S3 and Figure 4a,b). The microbial composition showed significant clustering by SCTLD stages in water and sediment samples (Table S4 and Figure 4c,d). While water samples showed discrete groupings, the sediment samples overlapped between the epidemic and endemic stages, with epidemic samples from Xesto Patch and Lindsay's Patch samples clustering closely. Cliff Green samples were more similar to one another, and endemic samples contributed to a lower *R* ^2^ value between epidemic and endemic sediment samples (*R* ^2^ = 0.07; Table S4 and Figure 4d).

### 3.6 The Disease Stage Had a Higher Explanatory Value of the Total Microbial Community Compared With Other Factors

The PERMANOVA analysis revealed significant interactions in structuring microbial communities, except for the relationship between season and date with coral species (Figure S2 and Table S5). In the coral samples, the largest explanatory factor was coral species (*R* ^2^ = 0.14), followed by disease stage (*R* ^2^ = 0.12). For water and sediment samples, disease stage was the strongest explanatory factor (*R* ^2^ = 0.45 for water and *R* ^2^ = 0.15 for sediment).

### 3.7 Random Forest Analysis Classified With High Accuracy the Three Disease Stages in Coral, Water, and Sediments

Random Forest Classification (RFC) analysis was performed within each sample type (coral, water, sediment) to find which ASVs best classify disease stages (vulnerable, epidemic, and endemic). For corals, ASVs tried for each split were set to a default of 31 out of 915, with a resample size = 74. The model's out‐of‐bag (OOB) performance metrics indicated a good classification performance (Brier score: 0.03, Normalised Brier score: 0.14, Area Under the Curve (AUC): 1, overall model misclassification (class) error: 0.01). In vulnerable samples, 26 samples were correctly classified by the model; 1 was misclassified (vulnerable class error = 0.04), and both epidemic and endemic samples had 0 samples misclassified (epidemic and endemic class errors = 0.0).

All water samples were correctly classified by the RFC model (class errors = 0.0), with 31 ASVs analysed in each split from 940 ASVs and a resample size of 61. The model's OOB performance metrics were also high for the water samples (Brier score: 0.01, Normalised Brier score: 0.03, AUC: 1, overall model misclassification error: 0.0).

For sediment samples, 67 ASVs were considered at each split from 4389, with a resample size of 57. The model's OOB performance metrics were also high (Brier score: 0.03, Normalised Brier score: 0.13, AUC: 1, overall model misclassification error: 0.01). The model accurately classified samples as vulnerable (vulnerable class error = 0.0) and epidemic (epidemic class error = 0.0), but had one misclassification in the endemic samples (endemic class error = 0.03). The ASVs with the highest model importance values in each sample type were selected (*n* = 30), and within each disease stage group, the top 10 from each were plotted (Figure S3).

### 3.8 Co‐Occurring Bacteria's Betweenness, Diameter, and Number of Neighbours in Coral, Water, and Sediment Changed With the SCTLD Stage

**FIGURE 5:**
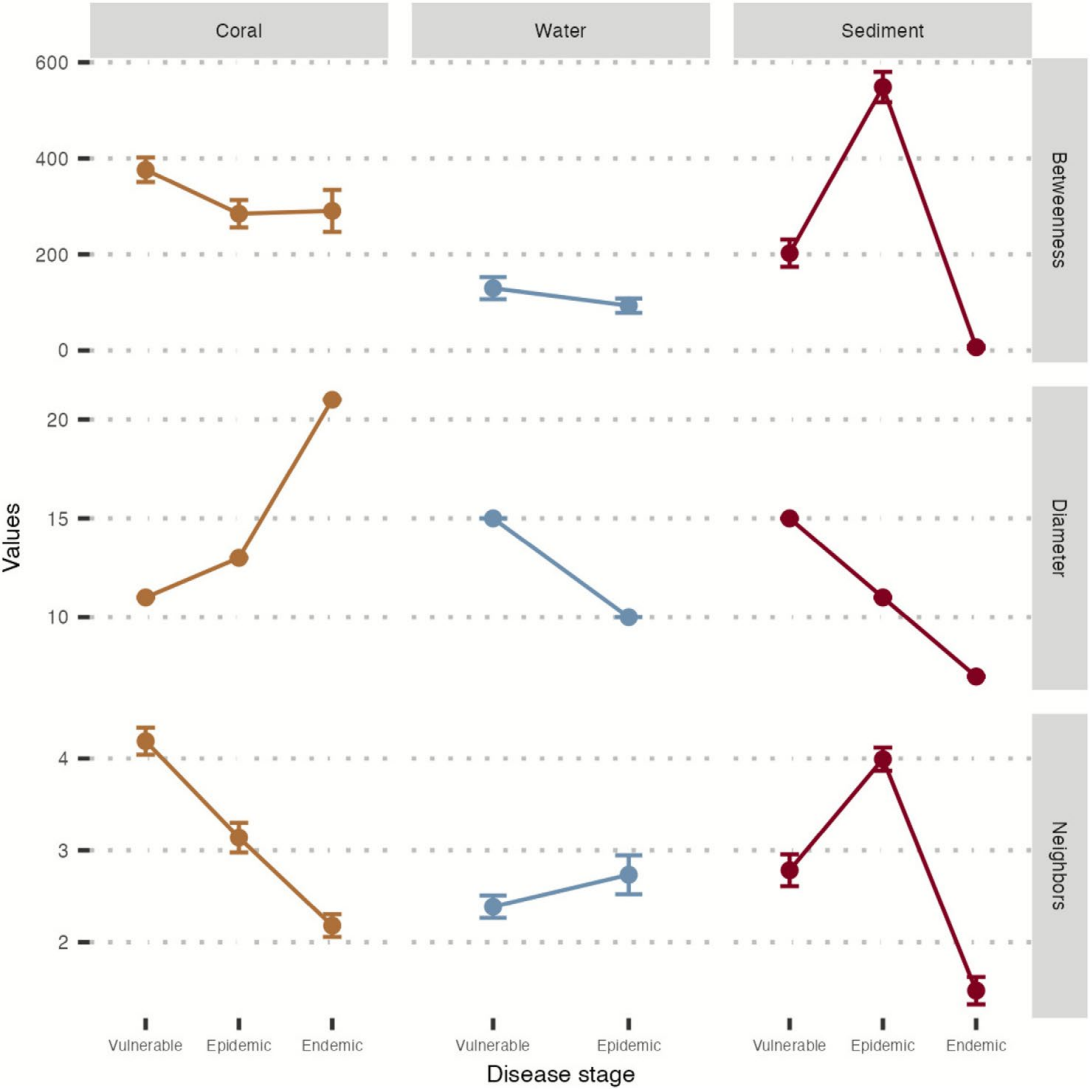
Network analysis indices of coral, water, and sediment across the three SCTLD stages. The figure is grouped and coloured by sample type, and network indices include: Betweenness (the number of shortest paths that pass through an edge, representing a bridge spanning role), diameter (the maximum length of the shortest path between any two connected nodes within the network), and neighbours (the number of adjacent edges or connections).

The network analysis revealed notable differences in various metrics (Figures 5 and S4, and Table S6). For corals, betweenness (the number of shortest paths that pass through an edge, representing a bridge‐spanning role) and neighbours (the number of adjacent edges or connections) significantly decreased in the epidemic and endemic stages compared with the vulnerable stage, but the network diameter (the maximum length of the shortest path between any two connected nodes within the network) was lower in the vulnerable stage compared with the epidemic and endemic stages. In water samples, measures of betweenness, network diameter, and neighbours significantly decreased from the vulnerable to epidemic stages (Figure 5 and Table S6), but our analysis failed to generate co‐occurring connections for the endemic disease stage in water samples. In sediment samples, betweenness and neighbours were significantly highest during the epidemic stage and lowest during the endemic stage. The network diameter in sediments mirrored the pattern observed in water samples: highest in the vulnerable stage and declining in the epidemic, and further during the endemic stage (Figures 5 and S4, Table S6).

### 3.9 Certain Important Bacterial Taxa (i.e., Key Players) Change in Relative Abundance Based on the SCTLD Stage

**FIGURE 6:**
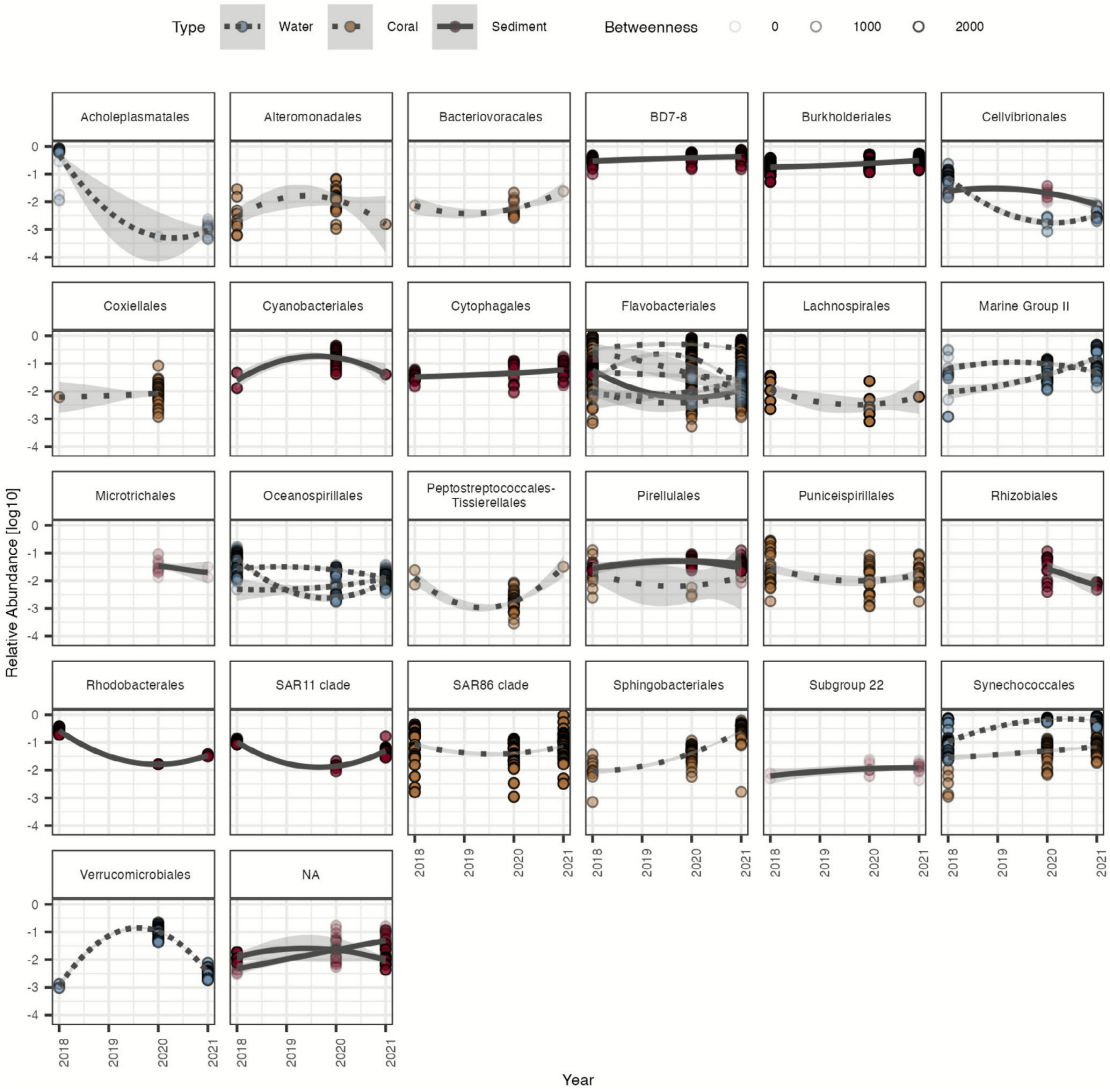
Relative abundance of nodes (i.e., bacteria) assigned as important in random forest and a key player in network analyses. The figure presents a line generated using a non‐parametric technique to create a smooth curve reflecting the relative abundance of each amplicon sequence variant (ASV). The grey shading around the line indicates a 95% confidence interval. Key players are categorised by taxonomic order, with ASVs within each order plotted together. Each ASV is assigned a unique colour and line pattern corresponding to the network in which it was identified as a key player (i.e., water, coral or sediment). The transition of the SCTLD stage is depicted by year, with 2018 representing vulnerable, 2020 as epidemic and 2021 as endemic stages.

Key players (taxa having the most impact on the network) identified in the network analysis demonstrated changes in relative abundance that may indicate shifts in disease stage (Figure 6). Focusing specifically on these key players, examining the relative abundance of each revealed notable trends: Altermonadales (vulnerable = 0.7% ± 0.9%, epidemic = 1.8% ± 1.9%, endemic = 0.2 ± NA) in corals, Verrucomicrobiales (vulnerable = 0.1% ± 0.1%, epidemic = 11.4% ± 5.0%, endemic = 0.1 ± 0.02) in water and Cyanobacteriales (vulnerable = 3.0% ± 2.4%, epidemic = 20.0% ± 11.2%, endemic = 4.1 ± NA) in sediments peaked in 2020 during the epidemic stage of SCTLD. Additionally, Coxiellales in corals (vulnerable = 0.5 ± NA, epidemic = 1.2% ± 1.5%, endemic = NA) and Rhizobiales (vulnerable = NA, epidemic = 4.0% ± 3.4%, endemic = 0.7% ± 0.3%) in sediments peaked in the epidemic disease stage and were absent in the endemic or vulnerable stage, respectively.

In contrast, in corals, the bacterial order Sphingobacteriales reached its peak in the endemic stage in 2021, with a mean relative abundance of 31% ± 13.2%, but it was still higher in the epidemic stage (4.9% ± 2.4%) compared with the vulnerable stage (1.4% ± 1.1%). Similarly, in corals, the order Synechococcales exhibited higher relative abundances in the epidemic (7.3% ± 10.1%) and endemic (8.4% ± 5.3%) stages compared with the vulnerable stage (5.1% ± 3.8%). This relative increase in Synechococcales in both the epidemic (67.8% ± 5.6%) and endemic (64.6% ± 18.2%) stages compared with the vulnerable stage (18.1% ± 22.1%) was also apparent in the water samples.

### 3.10 Coral and Sediments Both Showed Enrichment in Predicted Antibiotic Resistance Pathways in the Epidemic SCTLD Stage

PICRUSt2 analysis was used to infer the functional potential of microbial communities across the different disease stages. Differential abundance analysis identified significant KEGG Orthologies (KO) in coral (398), water (726), and sediment (1962) microbiomes (File S1). In coral microbiomes, epidemic and endemic stage communities showed significant enrichment (*p*adj ≤ 0.01) of KEGG degradation modules for salicylate, toluene, and xylene compared with the vulnerable stage (Figure S5a and File S1). Additionally, the multidrug resistance efflux pump MdtEF‐ToIC was significantly enriched (*p*adj ≤ 0.01) in the epidemic stage compared with the vulnerable stage (Figure S5a). Additional evidence for increased antimicrobial resistance potential during the epidemic stage was found in the identification of 10 cationic antimicrobial peptide (CAMP) resistance KOs, compared with only three in the endemic zone (File S1). In water samples, the only module showing at least a two‐fold log change was toluene degradation. The epidemic stage water samples had more significantly enriched KOs (394) than the endemic stage (332), but all at low coefficients (i.e., log‐fold changes below 2; Figure S5b and File S1). Similarly, sediment samples also exhibited more enriched KOs in the epidemic (1067) compared with the endemic stage (895). In both epidemic and endemic stages, sediments were enriched with carbapenem resistance modules that comprised the gene metallo‐beta‐lactamase class B (Figure S5c and File S1).

### 3.11 Predicated Pathways Were Enriched in Epidemic and Endemic Disease Stages

**FIGURE 7:**
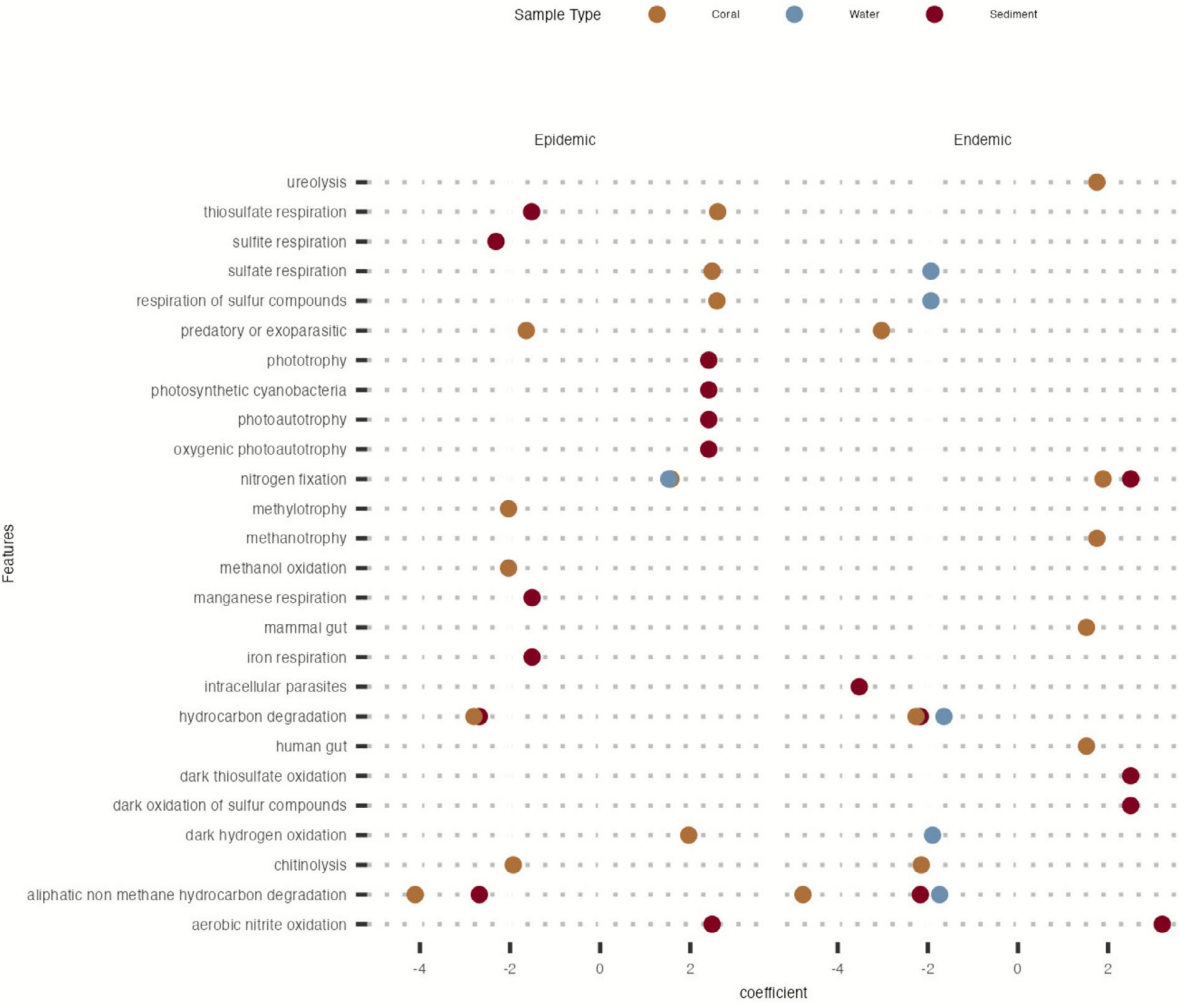
Significantly enriched putative metabolic pathways. Putative functional profiles via FAPROTAX were identified across three disease stages: vulnerable, epidemic and endemic. Each point represents a predicted putative function. The *x*‐axis indicates the direction and magnitude of association with disease stage. Positive coefficients denote features more abundant under the respective condition, while negative coefficients indicate depletion. A negative coefficient signifies enrichment in the vulnerable stage. Shown are functions with a *p*adj < 0.05, and coefficients (i.e., log fold change) between < −1.5 or > 1.5. Colours correspond to sample type (coral = brown, water = blue, sediment = burgundy).

The FAPROTAX results showed that coral samples had five enriched pathways during the epidemic stage, including three related to sulphur, sulphate and thiosulfate respiration (Figure 7). Both coral and water samples exhibited enrichment in nitrogen fixation; this was the only pathway enriched in water samples during the epidemic stage. Sediment samples had five enriched pathways at this stage, including four associated with phototrophy, photosynthetic cyanobacteria, and photoautotrophy.

During the endemic stage, coral microbiomes also displayed five enriched pathways, including ureolysis, methanotrophy, and nitrogen fixation, like in the epidemic stage. However, the taxonomic inference of functional potential showed that nitrogen fixation was not significant in water samples during the endemic stage but was enriched in sediments. Sediment samples had three other pathways enriched, which all included oxidation. No pathways were significant in water samples during the endemic stage.

### 3.12 SCTLD‐Associated Bacterial Richness and Relative Abundance Varied by Disease Stage Across Coral, Water, and Sediment

**FIGURE 8:**
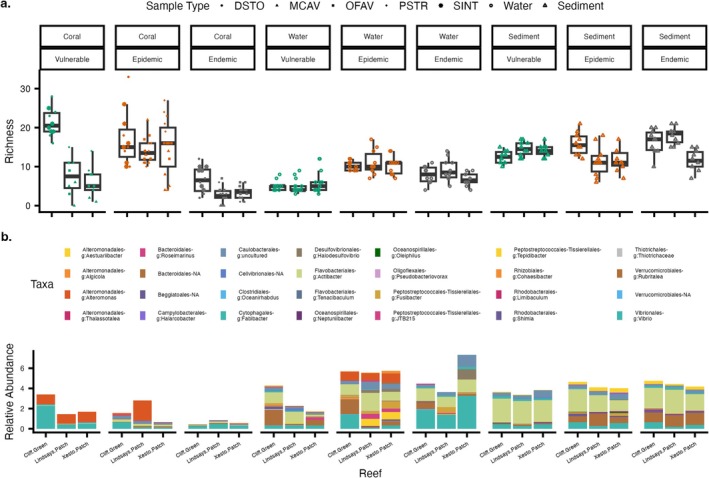
Detection of SCTLD‐associated amplicon sequence variants (ASVs) through disease stages in water, coral, and sediment at each of the three reefs. (a) Boxplots illustrate the richness (number) of SCTLD‐associated bacteria taxa detected at each reef. The sample points are coloured by SCTLD stages and shaped by coral species, sediment, or water. (b) Relative abundance of taxa identified in each sample type. The taxa are grouped and coloured by genus and organised by bacterial order.

Comparing ASVs from this study with candidate pathogens previously found enriched in SCTLD samples, we identified a total overlap of 82 ASVs (Figure 8a). When samples were grouped by reef, sample type, and disease stage, the highest mean richness (22.8 ± 3.0) of SCTLD‐associated bacteria was observed in coral samples, particularly within PSTR samples collected at Xesto Patch during the epidemic stage. This was closely followed by corals from Cliff Green, specifically DSTO (mean = 21.8 ± 4.8) and SINT (mean = 20.8 ± 2.5), during the vulnerable stage. Conversely, the lowest number of SCTLD‐associated taxa was recorded in MCAV samples from the endemic stage, with mean values of 0.8 ± 1.3 at Lindsay's Patch and 2.3 ± 1.5 at Xesto Patch. Notably, the mean of 2.3 for MCAV at Xesto Patch was the same as when it was in the vulnerable stage. In coral samples, all pairwise comparisons between epidemic and endemic were significantly different from one another (*p*adj ≥ 0.05; Table S7). Water samples from the vulnerable stage had the lowest average number of SCTLD‐associated taxa (5.4 ± 1.8), which increased significantly during the epidemic stage (10.6 ± 2.4) at Cliff Green (*p*adj ≥ 0.05) and Lindsay's Patch (*p*adj ≥ 0.05) before decreasing in the endemic stage (8.0 ± 2.4) to significantly lower levels than in the vulnerable stage at Lindsay's Patch (*p*adj ≥ 0.05; Table S7). In contrast, no significant change was found in SCTLD‐associated taxa in sediment samples, with mean values of 13.7 ± 1.9 for vulnerable, 13.0 ± 3.8 for epidemic, and 15.3 ± 3.8 for endemic stages (Table S7).

Although the number of SCTLD‐associated bacteria was low in corals during the endemic stage, some bacterial orders exhibited the highest relative abundances during this stage, including Vibrionales (30.9% ± 32.2%), Desulfovibrionales (20.2% ± 32.5%), Caulobacterales (17.7% ± 29.7%), Flavobacteriales (13.2% ± 13.5%) and Peptostreptococcales‐Tissierellales (11.2% ± 19.5%). Vulnerable water samples showed a low number of SCTLD‐associated taxa, but the highest abundances of Vibrionales (15.7% ± 11.5%) were found during the vulnerable stage compared with the epidemic (3.7% ± 2.6%; Figure 8b) and the endemic stages (4.1% ± 2.7%). Additionally, in water samples, Alteromonadales were high during the vulnerable (8.2% ± 6.0%) and epidemic stages (7.4% ± 16.6%), but declined during the endemic stage (0.7% ± 0.6%). In sediment samples, while there was little change in the number of taxa, the bacterial order Verrucomicrobiales was low during the vulnerable stage (1.4% ± 0.7%) before increasing during the epidemic stage (5.6% ± 3.8%) and remaining at a similar relative abundance in the endemic stage (5.2% ± 3.6%).

## 4 Discussion

This study examined coral reef microbial communities, as represented by coral, water and sediment, across time as they moved through the different stony coral tissue loss disease (SCTLD) stages: vulnerable, epidemic and endemic. This longitudinal sampling design controlled for the influence of geography, in comparison to previous SCTLD studies that examined contemporaneous samples from different reefs across the SCTLD stages. Thus, there may be random differences in the coral reef microbial communities as a result of the different sampling timepoints and seasons, independent of SCTLD stage. However, this approach can still provide insight into the several ways in which SCTLD stage can significantly influence the microbial diversity, composition and predicted functional potential of coral reef ecosystems by (1) considering the microbiome results in the context of the overall benthic habitat and coral community changes observed at Lower Keys patch reefs during and after SCTLD, (2) examining similarities in microbial response with previous SCTLD studies and (3) identifying the trends across stages of the specific microbial groups associated with SCTLD.

### 4.1 SCTLD Impacts Microbial Diversity in Apparently Healthy Corals Across Disease Stages

Across all coral species, alpha diversity and within‐group community variability (i.e., dispersion or beta diversity) significantly declined from the vulnerable to the endemic stage. These results suggest a reduction in microbial species richness, evenness, and variability in corals following SCTLD outbreaks. This indicates that even apparently healthy corals at reefs where SCTLD is endemic may be experiencing persistent effects of the disease at the microbial level. Interestingly, the vulnerable and epidemic stages mostly maintained similar alpha and beta diversity. Thus, the microbiomes of apparently healthy corals in the epidemic stage may not yet be significantly affected by the presence of SCTLD. This finding is consistent with the lack of significant differences between contemporaneous apparently healthy coral samples from the vulnerable and epidemic stages in DSTO (microbial groupings; Rosales et al. 2020), *Colpophyllia natans* , MCAV, OFAV, and *S. siderea* (beta diversity; Clark et al. 2021). However, by the endemic stage, corals likely had either been exposed to the disease or impacted by the overall ecosystem changes of the disease that led to an altered microbial community composition. For example, a decline in macroorganism (e.g., coral) diversity has been linked to ecosystem performance (Naeem et al. 1994), which could lead to a decrease in microbial diversity because of homogeneity in the environment (Curd et al. 2018). This effect would also be expected at our study sites following SCTLD, which reduced both the number of coral colonies and the species diversity at patch reefs in the Lower Keys, including one of our microbiome study sites, as indicated by the Coral Reef Evaluation and Monitoring Project (CREMP) data.

Higher alpha diversity among healthy corals during the vulnerable stage of SCTLD, compared with the epidemic and endemic stages, was observed previously but was not statistically significant (Rosales et al. 2023). A reduction in microbial community variability from the vulnerable to the epidemic stage of SCTLD among apparently healthy colonies has only been reported in PSTR (Clark et al. 2021). In this study, that pattern extended across coral species. Additionally, significant differences across SCTLD stages emerged in more individual species than in past studies, including MCAV and OFAV in addition to PSTR. This may reflect the strength of resampling the same reef sites, thereby controlling for geographical effects and likely enhancing the detection of within‐species patterns, but studies with more timepoints and that follow specific colonies are needed to better understand this result. DSTO and SINT displayed some of the most visually apparent declines across SCTLD stages in both diversity metrics, but these results were not significant. This may be due to the relatively lower sample sizes of these species, which were sampled from only Cliff Green. Regardless, these results highlight species‐specific differences in SCTLD response consistent with other microbial and gross assessments (Huntley et al. 2022; Precht et al. 2016).

### 4.2 A Fragmented Coral Microbial Community Network Showed Predicted Functional Changes in Nitrogen and Sulphur Cycle Dynamics Across Disease Stage

SCTLD may cause polymicrobial disturbances as indicated by the co‐occurrence network analysis. Similar to altered microbial connectivity in inflammatory bowel disease (Baldassano and Bassett 2016), coral networks in this study decreased in betweenness and neighbour counts during the epidemic and endemic stages. These changes indicate a more fragmented microbial network for apparently healthy corals within SCTLD‐affected reefs, aligning with declines in coral microbial diversity and alterations in community composition (Widder et al. 2014). The removal of bridge bacteria (i.e., bacteria that connect other bacteria) that stabilise the host microbiome (Peura et al. 2015) may impact microbial functional and metabolic pathways (Bissett et al. 2013).

PICRUSt2‐predicted enrichment analysis provided insights into how microbial communities might have changed by the SCTLD stage. The relatively increased abundance of potential multidrug‐resistant bacteria in corals during the epidemic stage suggests a shift toward antibiotic‐resistant bacteria and may be predicting a less cooperative microbial community, as antibiotic resistance is a form of microbial antagonism (Becker et al. 2012). We also observed a potential increase in predicted nitrogen fixation via the FAPROTAX analysis. Post‐SCTLD outbreak environmental conditions, such as enrichment in ammonium, correlated with increased heterotrophic bacteria like Flavobacteriaceae NS4 and NS5 marine groups (Becker et al. 2024). In this study, Flavobacteriales were identified as significant contributors in the random forest analysis and were 4 of 5 key players in the epidemic network analysis. This group has previously been enriched in apparently healthy corals at reefs in both the epidemic and endemic stages of SCTLD, as well as within non‐lesion (unaffected) tissue on corals with SCTLD (Rosales et al. 2023), highlighting their associations with corals under stress (McDevitt‐Irwin et al. 2017) and their potential to contribute to the hypothesized increase in nitrogen fixation genes.

The nitrogen‐fixing cyanobacteria Synechococcales (Esteves‐Ferreira et al. 2017) were also key players in the coral epidemic network analysis, and their relative abundance increased in corals in both epidemic and endemic stages. As coral reefs are typically nitrogen‐limited (Bell 1992), nitrogen‐fixing bacteria like Synechococcales play an important ecological role (Cardini et al. 2016). Synechococcales are enriched in apparently healthy corals from epidemic reefs, in healthy tissue of diseased corals (Rosales et al. 2023), and in diseased tissue (Heinz et al. 2024). Stressed corals may show higher levels of Synechococcales because corals consume them to relieve stress (Meunier et al. 2019; Radice et al. 2022). This pattern has been noted before in corals under thermal stress, which coincides with a higher abundance of Synechococcales in warmer weather (Phlips et al. 1999). However, our epidemic and endemic samples were collected during cooler weather (in February), suggesting that the increase in Synechococcales in stressed corals may not be temperature‐dependent. Instead, this study aligns with another study linking cyanobacteria to SCTLD (Becker et al. 2024) and other coral diseases (Cissell et al. 2022).

Excess nitrogen, such as ammonia (Wawrik et al. 2009), can stimulate Synechococcales and Cyanobacteriales. Urease genes found using metagenomes in SCTLD‐associated bacteria (Rosales et al. 2022) correspond with ammonia increases (Becker et al. 2024) and our observation of relatively increased Synechococcales abundances. Ureolysis—the lysis of urea to ammonia and carbamate (Mobley et al. 1995)—was a predicted function enriched in endemic stage corals and may contribute to increases in ammonia. Given the multiple studies showing a relationship between SCTLD and cyanobacteria, experiments should be conducted to understand if they mitigate or contribute to SCTLD development. Of importance, habitat surveys did not show an increase in benthic cyanobacteria during the epidemic and endemic stages in Lower Keys patch reefs, meaning that cyanobacteria may play a role in reef ecosystem dynamics even before any significant visual change, such as cyanobacterial mats.

Similarly, Sphingobacteriales were also more abundant in epidemic and endemic SCTLD stages. These bacteria, which play a key role in the decomposition process, may be central to the changes in the nitrogen cycle (Yan et al. 2024). During SCTLD, Sphingobacteriales may help degrade the increased organic matter, releasing nitrogen compounds that support enhanced nitrogen fixation. Previously, Sphingobacteriales were enriched in healthy tissue of diseased corals, but not in the lesions. Interestingly, enrichment of these bacteria was also only in reefs but not in tank experiments (Rosales et al. 2023), implying a role in reef nutrient cycling rather than being directly associated with the disease.

Solely in the epidemic stage, coral microbiomes exhibited predicted enrichment of sulphate metabolism pathways. Sulphide accumulation can cause Symbiodiniaceae to detach from coral tissue (Miller and Richardson 2012), and inhibiting sulphate‐reducing bacteria can prevent diseases like black band disease (BBD) (Brownell and Richardson 2014). Thiosulfate oxidation genes have been identified in putative pathogens associated with SCTLD and BBD, including members of Rhodobacterales, Rhizobiales, and Flavobacteriales (Bourne et al. 2013; Rosales et al. 2023). In this study, predicted pathways related to sulphate respiration were enriched, and we hypothesize SCTLD may have a comparative polymicrobial composition, as in BBD, composed of cyanobacteria, sulphate‐reducing bacteria, sulphide‐oxidising bacteria, and heterotrophic bacteria (Cooney et al. 2002).

### 4.3 Microbial Composition and Connectivity Changes in Water Samples From SCTLD‐Affected Reefs

Unlike coral samples, water samples showed increased microbiome variability from the vulnerable to the epidemic and endemic stages, suggesting that SCTLD impacts coral and environmental microbial communities differently. Furthermore, water samples did not differ in alpha diversity among cohorts, as coral samples did, but in the epidemic stage, betweenness and network diameter decreased compared with the vulnerable stage, possibly indicating reduced microbial connectivity (Baldassano and Bassett 2016). However, there was an increase in the neighbour connections. Although not significant, alpha diversity was higher in the epidemic compared with the vulnerable stage, which may reflect the increase in neighbours. In addition, the absence of a network for the endemic stage suggests a potential disruption in microbial interactions.

Verrucomicrobiales, identified as a putative SCTLD pathogen (Rosales et al. 2023), became a significant player in the epidemic stage of water networks. The relative increase in Verrucomicrobiales may have contributed to the decline in microbial betweenness in the epidemic water network by out‐competing other members through increased network connections. This group is broadly distributed in marine environments, may contribute to carbon cycling (Freitas et al. 2012), and may be attracted to diseased coral and invade necrotic coral tissue (Keller‐Costa et al. 2021).

In water samples, Synechococcales also emerged as a key player in the epidemic stage, and relatively increased in the epidemic and endemic stages compared with the vulnerable stage. Like others (Tout et al. 2014), we found that Synechococcales were relatively more abundant in the surrounding water column compared with corals (Tout et al. 2014). Because our epidemic‐stage water samples were collected above colonies with SCTLD, it may be that Synechococcales are attracted to the change in nutrient availability around diseased corals, making the water column a potential source of Synechococcales for coral heterotrophy (Meunier et al. 2019), as mentioned above. The putative increase in nitrification (nitrogen oxidation) modules for nitrate and nitrite synthesis in the water collected during the epidemic stage may be driven by an excess of ammonium in the environment, a possible change in nutrient availability related to diseased corals.

### 4.4 Decline in Sediment Microbial Community Variability and Increased Predicted Antibiotic Resistance in SCTLD‐Affected Reefs

In line with the water samples, sediments from the epidemic and endemic stages exhibited a significant increase in community variability compared with the vulnerable stage. Additionally, the overlap between the epidemic and endemic stage communities suggests limited change following the initial SCTLD impact. Also, compared with vulnerable stage sediments, sediments in the epidemic and endemic stages were enriched in bacteria with a potential for carbapenem resistance genes. Intriguingly, carbapenem resistance (a class of broad‐spectrum β‐lactam antibiotics) is associated with bacteria that resist carbapenem antibiotics—a class typically used to treat SCTLD (Neely et al. 2020; Forrester et al. 2022). As no antibiotic interventions occurred at these reefs (‘ArcGIS Dashboards’, n.d.), this predicted enrichment may indicate a natural microbial shift in response to SCTLD, with sediments potentially serving as a sink for antibiotic resistance genes after a disturbance (Guo et al. 2020; Zhang et al. 2018).

Co‐occurrence analysis revealed distinct differences between epidemic and endemic stage sediments. Epidemic stage networks were more connected and were dominated by Cyanobacteriales, Rhizobiales, and Burkholderiales, with the former two increasing in relative abundance. These networks also exhibited the highest betweenness and neighbour connectivity. While predicted nitrogen fixation was enriched only in endemic sediments, both stages showed enrichment in aerobic nitrite oxidation, potentially driven by Burkholderiales (Glaze et al. 2022). Cyanobacteria, the only oxygen‐producing nitrogen fixers, were linked to increased predicted phototrophy and photoautotrophy during the epidemic stage, highlighting their potential role in SCTLD dynamics across the reef environment. The emergence of Rhizobiales in the epidemic network is perhaps unsurprising in sediments collected near diseased corals (Rosales et al. 2023, 2020), suggesting microbial shedding from corals into surrounding sediments, where they can persist into the endemic stage of the disease (Rosales et al. 2020). Despite this persistence, Rhizobiales, along with the other key players of the epidemic stage network, do not sustain their interactions in the endemic stage of the disease, as indicated by the fragmented endemic network.

### 4.5 Persistent and Transient SCTLD‐Associated Bacteria in the Coral Reef Ecosystem

Our results show that some SCTLD‐associated bacteria identified in our meta‐analysis (Rosales et al. 2023) are present in non‐diseased corals and the reef environment across all SCTLD site stages, albeit in varying quantities. For coral samples, Cliff Green showed the highest number of SCTLD‐associated taxa within the vulnerable stage, which may, at least in part, be explained by seasonality. For example, Cliff Green corals were sampled in late June, when the sea surface temperature was 30.03°C—hotter relative to the May (27.04°C) and early June (28.70°C) sampling of Xesto and Lindsay's Patches. At the coral species level, the highest richness of SCTLD‐associated taxa associated with PSTR during the epidemic stage is perhaps expected, given that PSTR is highly susceptible to SCTLD (Papke et al. 2024), and apparently healthy corals sampled during the epidemic stage may be affected by the disease but not yet showing signs. Surprisingly, DSTO and SINT had the next highest richness of SCTLD‐associated bacteria in the vulnerable stage. This result may reflect site‐specific richness of these bacteria, since both coral species were only sampled at Cliff Green.

Additionally, Flavobacteriales and Verrucomicrobiales were also consistently abundant in Cliff Green corals across all stages. Given that corals can be affected by the disease before developing visible tissue loss lesions, this finding may suggest an initial microbial shift preceding gross signs of SCTLD (Landsberg et al. 2020). Time‐series sampling studies of apparently healthy corals at frequent intervals beginning in the vulnerable stage and progressing through the epidemic stage may help clarify some of these patterns.

The vulnerable stage water samples exhibited the lowest number of SCTLD taxa, which increased in the epidemic stage, potentially in relation to tissue shedding from diseased coral colonies. Still, they decreased in the endemic stage, suggesting that many of these bacteria are transient in the water column. This decrease further supports a relationship with SCTLD stage and not season, as epidemic and endemic sampling both occurred in February. In contrast, the high relative abundance of Alteromonadales and Vibrionales in the vulnerable stage indicates that sampling may have occurred in suboptimal water conditions for reefs. Indeed, vulnerable sampling for most of the water samples occurred during the summer months, a period known for higher levels of these bacteria due to warmer waters (McDevitt‐Irwin et al. 2017). Thus, the water column may be a source of Alteromonadales and Vibrionales, and under a disturbance, corals could attract these opportunists through chemotaxis (Tout et al. 2015). Altermonadales relatively increased in epidemic corals, while Vibrionales relatively increased in endemic corals. Although the number of SCTLD‐associated taxa appears to decline in endemic corals, the increased relative abundance of Vibrionales may imply that after SCTLD, Vibrionales become a more established community member, potentially making corals more susceptible to additional stressors, including SCTLD (Ushijima et al. 2020).

Sediments showed minimal changes in SCTLD‐associated taxa and may not serve as a source but rather a sink of these bacteria. For example, Verrucomicrobiales had a relative increase in the epidemic and endemic stages compared with the vulnerable stage, implying that sediments may act as a bioaccumulator for these bacteria following SCTLD disturbances (Hamamoto et al. 2024).

## 5 Conclusions

We show here that SCTLD not only causes alterations in benthic community composition through coral colony mortality and reductions in coral species diversity, but it also has lasting impacts on the coral reef ecosystem at the microbial level. Our study is limited because it is confounded by time, but it provides hypotheses of how a disease disturbance may alter reef microbial communities. By focusing our study on apparently healthy corals, along with reef water and sediments, at repeated sites as they moved through the stages of SCTLD, we have gained insight into the potential implications of the SCTLD disturbance on reef resilience. The observed decrease in diversity in the microbiomes of corals by the endemic stage of the disease mirrors the decreased coral species diversity caused by SCTLD, indicating negative lasting impacts on the health of remaining coral colonies. The fragmented microbial networks of both corals and sediment during the endemic stage of SCTLD show reduced connectivity that may compromise the stability and function of these ecosystems. The predicted enrichment of multidrug resistance and nitrogen/sulphur cycling highlights potential shifts in functional roles within microbial communities, raising concerns about antibiotic resistance and nutrient cycling within the ecosystem after an SCTLD disturbance. However, our functional prediction analyses serve as hypothesis generation and need to be verified with metagenomics and metatranscriptomics. SCTLD‐associated bacteria identified in the vulnerable stage are likely groups that are enriched in response to early disease exposure instead of acting as primary pathogens themselves. However, some taxa maintained high relative abundances in corals, water, and sediment in the endemic stage, indicating continued impacts from the disease. These results indicate that SCTLD may inhibit habitat recovery through feedback mechanisms between macro and micro species. In all, continuous monitoring of reef disturbances is needed to understand how disturbances can impact reef recovery.

## Author Contributions

Conceptualization: S.M.R., L.K.H., E.M.M. Data curation: S.M.R., G.J.K., A.S.C., L.K.H. Formal analysis: S.M.R. Funding acquisition: L.K.H., E.M.M., S.M.R. Investigation: S.M.R., L.K.H., A.S.C. Project administration: L.K.H. Resources: L.K.H., A.S.C. Visualisation: S.M.R. Writing – original draft preparation: S.M.R. Writing – reviewing and editing: All.

## Funding

This research was funded by an Environmental Protection Agency (EPA) 2019 South Florida Initiative grant (X7‐01D00820‐0) and carried out in part under the auspices of the Cooperative Institute for Marine and Atmospheric Studies, a cooperative institute of the University of Miami and the National Oceanic and Atmospheric Administration (NOAA), cooperative agreement NA 20OAR4320472.

## Conflicts of Interest

The authors declare no conflicts of interest.

## Supporting information


**Table S1:** Summary of sampling scheme. Samples are organised by collection date, with corresponding sea surface temperature (SST) values extracted from NOAA's jplMURSST41 database.
**Table S2:** Linear mixed models of Shannon diversity. Comparisons of microbial communities between three SCTLD stages (vulnerable, epidemic, and endemic) within three sample types: coral, sediment, and water. Significant adjusted values are in bold.
**Table S3:** Results of dispersed communities. Comparisons of microbial communities between three SCTLD stages (vulnerable, epidemic, and endemic) within three sample types: coral, sediment, and water. Bold values represent significant differences (*p* adjusted values) in community dispersion between the SCTLD stages within individual coral species *Dichocoenia stokesii* (DSTO), *Montastraea cavernosa* (MCAV), *Orbicella faveolata* (OFAV), *Pseudodiploria strigosa* (PSTR), and *Stephanocoenia intersepta* (SINT); across all coral species (‘Coral’); and across sediment and water samples.
**Table S4:** Results from PERMANOVA. Comparison of microbial community composition between the SCTLD stages within individual coral species *Dichocoenia stokesii* (DSTO), *Montastraea cavernosa* (MCAV), *Orbicella faveolata* (OFAV), *Pseudodiploria strigosa* (PSTR) and *Stephanocoenia intersepta* (SINT); across all coral species (‘Coral’); and sediment and water samples. Bolded values represent significant differences from *p* value adjusted results. The table also shows *R* ^2^ and *F* values.
**Table S5:** Results from PERMANOVA were used to identify significant interactions among various factors.
**Table S6:** Results from the Wilcoxon Rank Sum Test of network analysis attributes. Tests were conducted between SCTLD stages (vulnerable, epidemic, endemic) for coral, water, and sediment sample networks. The ‘*n*’ is the number of ASVs in each cohort.
**Table S7:** Results from the Wilcoxon Rank Sum Test of SCTLD‐Associated Bacteria Richness between reefs, disease stages, and sample types. Tests were conducted between SCTLD stages (vulnerable, epidemic, endemic) for coral, water, and sediment samples at each of the three reefs. The ‘*n*’ is the number of ASVs in each cohort.

**Figure S1:** Temporal trends in stony coral tissue loss disease (SCTLD) prevalence, benthic cover, coral counts, and live tissue area from 2014 to 2023 at Lower Keys patch reefs. (a) Monthly counts of SCTLD‐affected coral colonies at random sites from the Disturbance Response Monitoring (DRM) program. From four repeated‐measures sites in the Coral Reef Evaluation and Monitoring Project (CREMP), surveyed in the summer: (b) Percent cover of benthic groups, including macroalgae, cyanobacteria, stony corals, bare substrate and others, shown as mean annual percent cover ± SE for each group. (c) Total colony counts and (d) total living tissue area (LTA, cm^2^) for the five focal SCTLD‐susceptible coral species examined in this study (*Dichocoenia stokesii*, *Montastraea cavernosa* , *Orbicella* spp., *Pseudodiploria strigosa* and *Stephanocoenia intersepta* ), aggregated annually across CREMP sites. (d) Total colony counts and (f) LTA for seven other SCTLD‐susceptible or common coral species: *Colpophyllia natans* , *Diploria labyrinthiformis* , *Eusmilia fastigiata* , *Meandrina meandrites* , *Mycetophyllia* spp., *Porites astreoides* and *Siderastrea siderea* . Shaded grey bars in all panels denote microbiome sampling time: the vulnerable stage (May–June 2018), epidemic stage (February 2020) and endemic stage (February 2021).

**Figure S2:** Factors and their interactions were tested for explanatory value in structuring microbial communities. Shown are the *R* ^2^ values of each factor or interaction, with bubbles scaled to *p* values (the bigger the bubble, the smaller the *p* value), tested with PERMANOVA. ‘Coral species’ were *Dichocoenia stokesii* (DSTO), *Stephanocoenia intersepta* (SINT), *Montastraea cavernosa* (MCAV), *Pseudodiploria strigosa* (PSTR) and *Orbicella faveolata* (OFAV); ‘Date’ is sampling date (Figure 1); ‘Season’ represents wet or dry season (Table S1); ‘Reef’ represents three Lower Keys patch reefs (Figure 1); ‘Project’ refers to whether the samples came from Rosales et al. (2020), Clark et al. (2021) or new collections for this study; and ‘Disease stage’ is vulnerable, epidemic or endemic.

**Figure S3:** The top 10 bacteria taxa per SCTLD stage and sample type, with the highest importance values assigned by random forest analysis. Bacteria amplicon sequence variants (ASVs) are coloured based on whether they were assigned as important in water, coral or sediment random forest analysis. The overall ASV importance is displayed on the *x*‐axis and grouped by disease stage. The *y*‐axis is grouped by bacteria order and then by genus. The bubble size indicates the importance of the ASV for each disease stage (i.e., vulnerable, epidemic or endemic).

**Figure S4:** Network analysis across SCTLD disease stages for (a) coral, (b) water, and (c) sediment. Nodes represent amplicon sequence variants (ASVs), with their size indicating the number of neighbouring ASVs. A triangle and label identifying the bacterial order signify that a node is a key player in the network. The width of the edges (i.e., the lines connecting the nodes) reflects centrality, with thicker edges indicating higher centrality. The alpha transparency of each point represents the overall importance of the random forest model.

**Figure S5:** Heatmaps show the predicted functions of the microbiome community for KEGG pathways of (a) coral, (b) water, and (c) sediments. The columns represent the disease stages, endemic and epidemic, relative to the vulnerable stage. The rows indicate different KEGG orthologies (KOs) and are grouped by modules. A negative coefficient or log fold change signifies enrichment in the vulnerable stage.

**File S1:** Statistical outputs of differential abundance analysis of predicted functions of the microbiome community for KEGG pathways. The file provides the features tested for significance, the sample types, and the significance values.

## Data Availability

The code, count data, taxonomy data, and ASVs sequences are available on https://github.com/srosales712/SCTLD‐zones. The data are available on NCBI under BioProject numbers PRJNA576217, PRJNA1281705, and PRJNA772194.
