# Supplementary material for: Stony Coral Tissue Loss Disease Results in Persistent Microbial‐Level Disturbances on Coral Reef Ecosystems
Source: Environ Microbiol Rep. 2025 Dec 21;17(6):e70264. doi: 10.1111/1758-2229.70264 (PMC12719614; doi:10.1111/1758-2229.70264)
Supplement: Supplementary file 1 — Table S1: Summary of sampling scheme. Samples are organised by collection date, with corresponding sea surface temperature (SST) values extracted from NOAA's jplMURSST41 database. Table S2: Linear mixed models of Shannon diversity. Comparisons of microbial communities between three SCTLD stages (vulnerable, epidemic, and endemic) within three sample types: coral, sediment, and water. Significant adjusted values are in bold. Table S3: Results of dispersed communities. Comparisons of microbial communities between three SCTLD stages (vulnerable, epidemic, and endemic) within three sample types: coral, sediment, and water. Bold values represent significant differences (p adjusted values) in community dispersion between the SCTLD stages within individual coral species Dichocoenia stokesii (DSTO), Montastraea cavernosa (MCAV), Orbicella faveolata (OFAV), Pseudodiploria strigosa (PSTR), and Stephanocoenia intersepta (SINT); across all coral species (‘Coral’); and across sediment and water samples. Table S4: Results from PERMANOVA. Comparison of microbial community composition between the SCTLD stages within individual coral species Dichocoenia stokesii (DSTO), Montastraea cavernosa (MCAV), Orbicella faveolata (OFAV), Pseudodiploria strigosa (PSTR) and Stephanocoenia intersepta (SINT); across all coral species (‘Coral’); and sediment and water samples. Bolded values represent significant differences from p value adjusted results. The table also shows R 2 and F values. Table S5: Results from PERMANOVA were used to identify significant interactions among various factors. Table S6: Results from the Wilcoxon Rank Sum Test of network analysis attributes. Tests were conducted between SCTLD stages (vulnerable, epidemic, endemic) for coral, water, and sediment sample networks. The ‘n’ is the number of ASVs in each cohort. Table S7: Results from the Wilcoxon Rank Sum Test of SCTLD‐Associated Bacteria Richness between reefs, disease stages, and sample types. Tests were conducted betw [file EMI4-17-e70264-s002.docx]

Supplemental Tables

Stony coral tissue loss disease results in persistent microbial-level disturbances on coral reef ecosystems

Stephanie M. Rosales^1,2^, J. Grace Klinges^3,4^, Abigail S. Clark^4,5^, Erinn M. Muller^6^, Lindsay K. Huebner^7^

1. Cooperative Institute for Marine and Atmospheric Studies, University of Miami, Miami, FL, United States

2. Atlantic Oceanographic and Meteorological Laboratory, National Oceanic and Atmospheric Administration, Miami, FL, United States

3. Center for Global Discovery and Conservation Science, Arizona State University, Hilo, HI, USA

4. Elizabeth Moore International Center for Coral Reef Research & Restoration, Mote Marine Laboratory, Summerland Key, FL, USA

5. Scouting America, Sea Base, Brinton Environmental Center, Summerland Key, FL, USA

6. Mote Marine Laboratory, Sarasota, FL, USA

7. Fish and Wildlife Research Institute, Florida Fish and Wildlife Conservation Commission, St. Petersburg, FL, United States

**Supplementary Table 1. Summary of sampling scheme**. Samples are organized by collection date, with corresponding sea surface temperature (SST) values extracted from NOAA’s jplMURSST41 database.

| **Reef** | **Type** | **Site**  **Stage** | **Sample Date** | **Latitude** | **Longitude** | **SST ℃** | **Season** |
| --- | --- | --- | --- | --- | --- | --- | --- |
| Xesto Patch | Coral | Vulnerable | 5/8/18 | 24.50156 | -81.74487 | 27.053 | Dry |
| Xesto Patch | Water | Vulnerable | 5/8/18 | 24.501657 | -81.745887 | 27.053 | Dry |
| Lindsays Patch | Coral | Vulnerable | 6/5/18 | 24.51244 | -81.78568 | 28.699 | Wet |
| Lindsays  Patch | Water | Vulnerable | 6/5/18 | 24.512557 | -81.785876 | 28.699 | Wet |
| Cliff Green | Coral | Vulnerable | 6/28/18 | 24.5036 | -81.7677 | 30.029 | Wet |
| Cliff Green | Sediment | Vulnerable | 6/28/18 | 24.5036 | -81.7677 | 30.029 | Wet |
| Cliff Green | Water | Vulnerable | 6/28/18 | 24.5036 | -81.7677 | 30.029 | Wet |
| Xesto Patch | Sediment | Vulnerable | 6/28/18 | 24.501657 | -81.745887 | 30.059 | Wet |
| Xesto Patch | Water | Vulnerable | 6/28/18 | 24.501657 | -81.745887 | 30.059 | Wet |
| Lindsays Patch | Sediment | Vulnerable | 6/28/18 | 24.512557 | -81.785876 | 30.127 | Wet |
| Lindsays  Patch | Water | Vulnerable | 6/28/18 | 24.512557 | -81.785876 | 30.127 | Wet |
| Xesto Patch | Coral | Epidemic | 2/11/20 | 24.501657 | -81.745887 | 25.036 | Dry |
| Xesto Patch | Sediment | Epidemic | 2/11/20 | 24.501657 | -81.745887 | 25.036 | Dry |
| Xesto Patch | Water | Epidemic | 2/11/20 | 24.501657 | -81.745887 | 25.036 | Dry |
| Lindsays Patch | Coral | Epidemic | 2/12/20 | 24.512557 | -81.785876 | 24.479 | Dry |
| Lindsays Patch | Sediment | Epidemic | 2/12/20 | 24.512557 | -81.785876 | 24.479 | Dry |
| Lindsays Patch | Water | Epidemic | 2/12/20 | 24.512557 | -81.785876 | 24.479 | Dry |
| Cliff Green | Sediment | Epidemic | 2/12/20 | 24.5036 | -81.7677 | 24.509 | Dry |
| Cliff Green | Water | Epidemic | 2/12/20 | 24.5036 | -81.7677 | 24.509 | Dry |
| Xesto Patch | Coral | Epidemic | 2/13/20 | 24.501657 | -81.745887 | 24.016 | Dry |
| Lindsays  Patch | Coral | Epidemic | 2/13/20 | 24.512557 | -81.785876 | 24.479 | Dry |
| Cliff Green | Coral | Epidemic | 2/14/20 | 24.5036 | -81.7677 | 24.287 | Dry |
| Xesto Patch | Coral | Endemic | 2/12/21 | 24.501657 | -81.745887 | 23.046 | Dry |
| Xesto Patch | Sediment | Endemic | 2/12/21 | 24.501657 | -81.745887 | 23.046 | Dry |
| Xesto Patch | Water | Endemic | 2/12/21 | 24.501657 | -81.745887 | 23.046 | Dry |
| Cliff Green | Coral | Endemic | 2/12/21 | 24.5036 | -81.7677 | 23.164 | Dry |
| Lindsays  Patch | Coral | Endemic | 2/12/21 | 24.512557 | -81.785876 | 23.321 | Dry |
| Lindsays  Patch | Sediment | Endemic | 2/12/21 | 24.512557 | -81.785876 | 23.321 | Dry |
| Lindsays  Patch | Water | Endemic | 2/12/21 | 24.512557 | -81.785876 | 23.321 | Dry |
| Lindsays  Patch | Sediment | Endemic | 2/13/21 | 24.512557 | -81.785876 | 23.321 | Dry |
| Lindsays  Patch | Water | Endemic | 2/13/21 | 24.512557 | -81.785876 | 23.321 | Dry |
| Cliff Green | Sediment | Endemic | 2/13/21 | 24.5036 | -81.7677 | 24.38 | Dry |
| Cliff Green | Water | Endemic | 2/13/21 | 24.5036 | -81.7677 | 24.38 | Dry |

**Supplementary Table 2. Linear mixed models of Shannon diversity.** Comparisons of microbial communities between three SCTLD stages (Vulnerable, Epidemic, and Endemic) within three sample types: coral, sediment, and water. Significant adjusted values are in bold.

| **SCTLD stage** | **Estimate** | **Std. Error** | **z value** | **Pr(>\|z\|)** | **Sample type** |
| --- | --- | --- | --- | --- | --- |
| Epidemic - Vulnerable | 0.1333 | 0.2921 | 0.456 | 0.8883 | Coral |
| Endemic - Vulnerable | -0.7057 | 0.2888 | -2.444 | **0.0364** | Coral |
| Endemic - Epidemic | -0.8390 | 0.1666 | -5.035 | **<1e-04** | Coral |
| Epidemic - Vulnerable | 0.29862 | 0.05637 | 5.298 | **< 1e-04** | Sediment |
| Endemic - Vulnerable | 0.52316 | 0.05637 | 9.282 | **< 1e-04** | Sediment |
| Endemic - Epidemic | 0.22455 | 0.05637 | 3.984 | **0.000201** | Sediment |
| Epidemic - Vulnerabl | -0.03247 | 0.10310 | -0.315 | 0.9439 | Water |
| Endemic - Vulnerable | -0.12814 | 0.10310 | -1.243 | 0.4102 | Water |
| Endemic - Epidemic | -0.09567 | 0.04318 | -2.216 | 0.0622 | Water |

**Supplementary Table 3. Results of dispersed communities.** Comparisons of microbial communities between three SCTLD stages (Vulnerable, Epidemic, and Endemic) within three sample types: coral, sediment, and water. Bold values represent significant differences (p-adjusted values) in community dispersion between the SCTLD stages within individual coral species *Dichocoenia stokesii* (DSTO), *Montastraea cavernosa* (MCAV), *Orbicella faveolata* (OFAV), *Pseudodiploria strigosa* (PSTR), and *Stephanocoenia intersepta* (SINT); across all coral species (‘Coral’); and across sediment and water samples.

| **SCTLD stage** | **DSTO** | **MCAV** | **OFAV** | **PSTR** | **SINT** | **Coral** | **Sediment** | **Water** |
| --- | --- | --- | --- | --- | --- | --- | --- | --- |
| **Epidemic-**  **Vulnerable** | 0.86538 | 0.88538 | **0.03748** | 0.96412 | 0.93323 | 0.07009 | **0.0000** | **0.001739** |
| **Endemic-**  **Vulnerable** | 0.33601 | 0.08039 | 0.91030 | **0.04151** | 0.10246 | **0.00000** | **0.0000** | **0.000036** |
| **Endemic-**  **Epidemic** | 0.14931 | **0.00982** | **0.03213** | **0.02147** | 0.16023 | **0.00017** | 0.99934 | 0.561356 |

**Supplementary Table 4. Results from PERMANOVA**. Comparison of microbial community composition between the SCTLD stages within individual coral species *Dichocoenia stokesii* (DSTO), *Montastraea cavernosa* (MCAV), *Orbicella faveolata* (OFAV), *Pseudodiploria strigosa* (PSTR), and *Stephanocoenia intersepta* (SINT); across all coral species (‘Coral’); and sediment and water samples. Bolded values represent significant differences from pvalue adjusted results. The table also shows R^2^ and F. values.

| **SCTLD stage** | **Statistic** | **DSTO** | **MCAV** | **OFAV** | **PSTR** | **SINT** | **Coral** | **Sediment** | **Water** |
| --- | --- | --- | --- | --- | --- | --- | --- | --- | --- |
| Epidemic-Vulnerable | padj | **0.009** | **0.006** | **0.003** | **0.003** | **0.009** | **0.003** | **0.003** | **0.003** |
|  | R^2^ | 0.33 | 0.18 | 0.19 | 0.19 | 0.40 | 0.09 | 0.14 | 0.47 |
|  | F-value | 4.37 | 3.08 | 3.47 | 2.79 | 5.90 | 7.46 | 10.10 | 57.32 |
| Endemic-  Vulnerable | padj | **0.033** | **0.003** | **0.003** | **0.003** | **0.009** | **0.003** | **0.003** | **0.003** |
|  | R^2^ | 0.01 | 0.16 | 0.17 | 0.16 | 0.37 | 0.10 | 0.13 | 0.40 |
|  | F-value | 3.59 | 3.11 | 3.16 | 2.84 | 5.37 | 7.14 | 8.62 | 42.11 |
| Endemic-  Epidemic | padj | **0.006** | **0.003** | **0.003** | **0.003** | **0.009** | **0.003** | **0.003** | **0.003** |
|  | R^2^ | 0.180 | 0.16 | 0.16 | 0.13 | 0.16 | 0.09 | 0.07 | 0.22 |
|  | F-value | 1.98 | 3.80 | 4.08 | 3.05 | 2.00 | 8.39 | 4.58 | 16.09 |

**Supplementary Table 5. Results from PERMANOVA were used to identify significant interactions among various factors.**

| **Factor** | **Df** | **SumOfSqs** | **MeanSqs** | **F.Model** | **R²** | **Pr(>F)** | **Sample Type** |
| --- | --- | --- | --- | --- | --- | --- | --- |
| **Disease Stage** | 2 | 70058 | 35029 | 10.4248 | 0.12 | 0.001 *** | Coral |
| **Season** | 1 | 22363 | 22363 | 6.6552 | 0.04 | 0.001 *** | Coral |
| **Coral Species** | 4 | 80917 | 20229 | 6.0203 | 0.14 | 0.001 *** | Coral |
| **Project** | 1 | 16417 | 16417 | 4.8857 | 0.03 | 0.001 *** | Coral |
| **Reef** | 1 | 6085 | 6085 | 1.8110 | 0.01 | 0.009 ** | Coral |
| **Date** | 3 | 19033 | 6344 | 1.8881 | 0.03 | 0.004 ** | Coral |
| **Disease Stage:**  **Coral Species** | 6 | 41517 | 6920 | 2.0593 | 0.07 | 0.001 *** | Coral |
| **Season:**  **Coral Species** | 2 | 8250 | 4125 | 1.2276 | 0.01 | 0.125 | Coral |
| **Coral Species:**  **Reef** | 2 | 8703 | 4352 | 1.2951 | 0.01 | 0.046 * | Coral |
| **Coral Species:**  **Date** | 2 | 8311 | 4155 | 1.2367 | 0.01 | 0.084 | Coral |
| **Season** | 1 | 11864 | 11864 | 11.528 | 0.05 | 0.001 *** | Water |
| **Project** | 1 | 9236 | 9236 | 8.974 | 0.04 | 0.001 *** | Water |
| **Reef** | 2 | 7726 | 3863 | 3.754 | 0.03 | 0.001 *** | Water |
| **Date** | 2 | 17762 | 8881 | 8.629 | 0.07 | 0.001 *** | Water |
| **Disease Stage:**  **Reef** | 3 | 11612 | 3871 | 3.761 | 0.04 | 0.001 *** | Water |
| **Site Stage** | 2 | 170910 | 85455 | 8.6539 | 0.15 | 0.001 *** | Sediment |
| **Reef** | 2 | 90827 | 45413 | 4.5989 | 0.08 | 0.001 *** | Sediment |
| **Date** | 2 | 33445 | 16723 | 1.6935 | 0.03 | 0.009 ** | Sediment |
| **Disease Stage:**  **Reef** | 3 | 69889 | 23296 | 2.3592 | 0.06 | 0.001 *** | Sediment |

**Supplementary Table 6. Results from the Wilcoxon Rank Sum Test of network analysis attributes**. Tests were conducted between SCTLD stages (vulnerable, epidemic, endemic) for coral, water, and sediment sample networks. The “n” is the number of ASVs in each cohort.

| **Sample type** | **Network attribute** | **SCTLD stage 1** | **SCTLD stage 2** | **n1** | **n2** | **stat** | **p** | **p.adj** | **P.adj**  **significance** |
| --- | --- | --- | --- | --- | --- | --- | --- | --- | --- |
| Coral | Between-ness | Vulnerable | Epidemic | 211 | 144 | 18005 | 3.00e-03 | 1.2000e-02 | * |
| Coral | Between-ness | Vulnerable | Endemic | 211 | 122 | 16974 | 1.10e-06 | 8.8000e-06 | **** |
| Coral | Between-ness | Epidemic | Endemic | 144 | 122 | 10190 | 2.20e-02 | 4.5000e-02 | * |
| Sediment | Between-ness | Vulnerable | Epidemic | 120 | 295 | 9145 | 1.01e-14 | 1.6160e-13 | **** |
| Sediment | Between-ness | Vulnerable | Endemic | 120 | 53 | 5135 | 2.73e-11 | 4.0950e-10 | **** |
| Sediment | Between-ness | Epidemic | Endemic | 295 | 53 | 14422 | 8.13e-23 | 1.7073e-21 | **** |
| Water | Between-ness | Vulnerable | Epidemic | 135 | 87 | 8663 | 1.83e-09 | 2.5620e-08 | **** |
| Coral | neighbors | Vulnerable | Epidemic | 211 | 144 | 19948 | 3.96e-07 | 3.5640e-06 | **** |
| Coral | neighbors | Vulnerable | Endemic | 211 | 122 | 20403 | 1.90e-19 | 3.8000e-18 | **** |
| Coral | neighbors | Epidemic | Endemic | 144 | 122 | 11561 | 5.10e-06 | 3.5700e-05 | **** |
| Sediment | neighbors | Vulnerable | Epidemic | 120 | 295 | 11452 | 1.11e-08 | 1.2210e-07 | **** |
| Sediment | neighbors | Vulnerable | Endemic | 120 | 53 | 4895 | 3.70e-09 | 4.8100e-08 | **** |
| Sediment | neighbors | Epidemic | Endemic | 295 | 53 | 13727 | 7.39e-19 | 1.4041e-17 | **** |
| Water | neighbors | Vulnerable | Epidemic | 135 | 87 | 6860 | 3.20e-02 | 4.5000e-02 | * |
| All | Diameter | Vulnerable | Epidemic | 466 | 526 | 144257 | 1.52e-08 | 1.52e-08 | **** |
| All | Diameter | Vulnerable | Endemic | 466 | 175 | 24698 | 2.80e-17 | 8.40e-17 | **** |
| All | Diameter | Epidemic | Endemic | 526 | 175 | 27878 | 2.38e-16 | 4.76e-16 | **** |

**Supplementary Table 7. Results from the Wilcoxon Rank Sum Test of SCTLD-Associated Bacteria Richness between Reefs, Disease stages, and Sample types**. Tests were conducted between SCTLD stages (vulnerable, epidemic, endemic) for coral, water, and sediment samples at each of the three reefs. The “n” is the number of ASVs in each cohort.

| **Reef** | **Sample**  **type** | **Alpha**  **Diversity** | **SCTLD stage 1** | **SCTLD stage 2** | **n1** | **n2** | **stat** | **p** | **p.adj** | **P.adj**  **significance** |
| --- | --- | --- | --- | --- | --- | --- | --- | --- | --- | --- |
| Cliff Green | Coral | Richness | Vulnerable | Epidemic | 10 | 11 | 83.5 | 0.048 | 1 | ns |
| Cliff Green | Coral | Richness | Vulnerable | Endemic | 10 | 12 | 120 | 8.57E-05 | 0.0023139 | ** |
| Cliff Green | Coral | Richness | Epidemic | Endemic | 11 | 12 | 127 | 1.87E-04 | 0.005049 | ** |
| Cliff Green | Water | Richness | Vulnerable | Epidemic | 10 | 10 | 0 | 1.51E-04 | 0.004077 | ** |
| Cliff Green | Water | Richness | Vulnerable | Endemic | 10 | 10 | 15 | 0.008 | 0.216 | ns |
| Cliff Green | Water | Richness | Epidemic | Endemic | 10 | 10 | 79 | 0.028 | 0.756 | ns |
| Cliff Green | Sediment | Richness | Vulnerable | Epidemic | 10 | 10 | 14.5 | 0.008 | 0.216 | ns |
| Cliff Green | Sediment | Richness | Vulnerable | Endemic | 10 | 10 | 14 | 0.007 | 0.189 | ns |
| Cliff Green | Sediment | Richness | Epidemic | Endemic | 10 | 10 | 43 | 0.621 | 1 | ns |
| Lindsays Patch | Coral | Richness | Vulnerable | Epidemic | 8 | 16 | 16 | 0.003 | 0.081 | ns |
| Lindsays Patch | Coral | Richness | Vulnerable | Endemic | 8 | 18 | 117.5 | 0.011 | 0.297 | ns |
| Lindsays Patch | Coral | Richness | Epidemic | Endemic | 16 | 18 | 288 | 6.60E-07 | 1.78E-05 | *** |
| Lindsays Patch | Water | Richness | Vulnerable | Epidemic | 13 | 10 | 4.5 | 1.67E-04 | 0.004509 | ** |
| Lindsays Patch | Water | Richness | Vulnerable | Endemic | 13 | 10 | 13 | 0.001 | 0.027 | * |
| Lindsays Patch | Water | Richness | Epidemic | Endemic | 10 | 10 | 66 | 0.238 | 1 | ns |
| Lindsays Patch | Sediment | Richness | Vulnerable | Epidemic | 10 | 10 | 77.5 | 0.04 | 1 | ns |
| Lindsays Patch | Sediment | Richness | Vulnerable | Endemic | 10 | 10 | 13 | 0.005 | 0.135 | ns |
| Lindsays Patch | Sediment | Richness | Epidemic | Endemic | 10 | 10 | 8 | 0.002 | 0.054 | ns |
| Xesto Patch | Coral | Richness | Vulnerable | Epidemic | 9 | 15 | 22.5 | 0.008 | 0.216 | ns |
| Xesto Patch | Coral | Richness | Vulnerable | Endemic | 9 | 18 | 119.5 | 0.048 | 1 | ns |
| Xesto Patch | Coral | Richness | Epidemic | Endemic | 15 | 18 | 254 | 1.60E-05 | 4.32E-04 | *** |
| Xesto Patch | Water | Richness | Vulnerable | Epidemic | 13 | 10 | 19.5 | 0.005 | 0.135 | ns |
| Xesto Patch | Water | Richness | Vulnerable | Endemic | 13 | 10 | 43.5 | 0.184 | 1 | ns |
| Xesto Patch | Water | Richness | Epidemic | Endemic | 10 | 10 | 88.5 | 0.004 | 0.108 | ns |
| Xesto Patch | Sediment | Richness | Vulnerable | Epidemic | 10 | 10 | 80.5 | 0.022 | 0.594 | ns |
| Xesto Patch | Sediment | Richness | Vulnerable | Endemic | 10 | 10 | 77 | 0.042 | 1 | ns |
| Xesto Patch | Sediment | Richness | Epidemic | Endemic | 10 | 10 | 49 | 0.97 | 1 | ns |
