# Supplementary figures and images for: Stony Coral Tissue Loss Disease Results in Persistent Microbial‐Level Disturbances on Coral Reef Ecosystems
Source: Environ Microbiol Rep. 2025 Dec 21;17(6):e70264. doi: 10.1111/1758-2229.70264 (PMC12719614; doi:10.1111/1758-2229.70264)

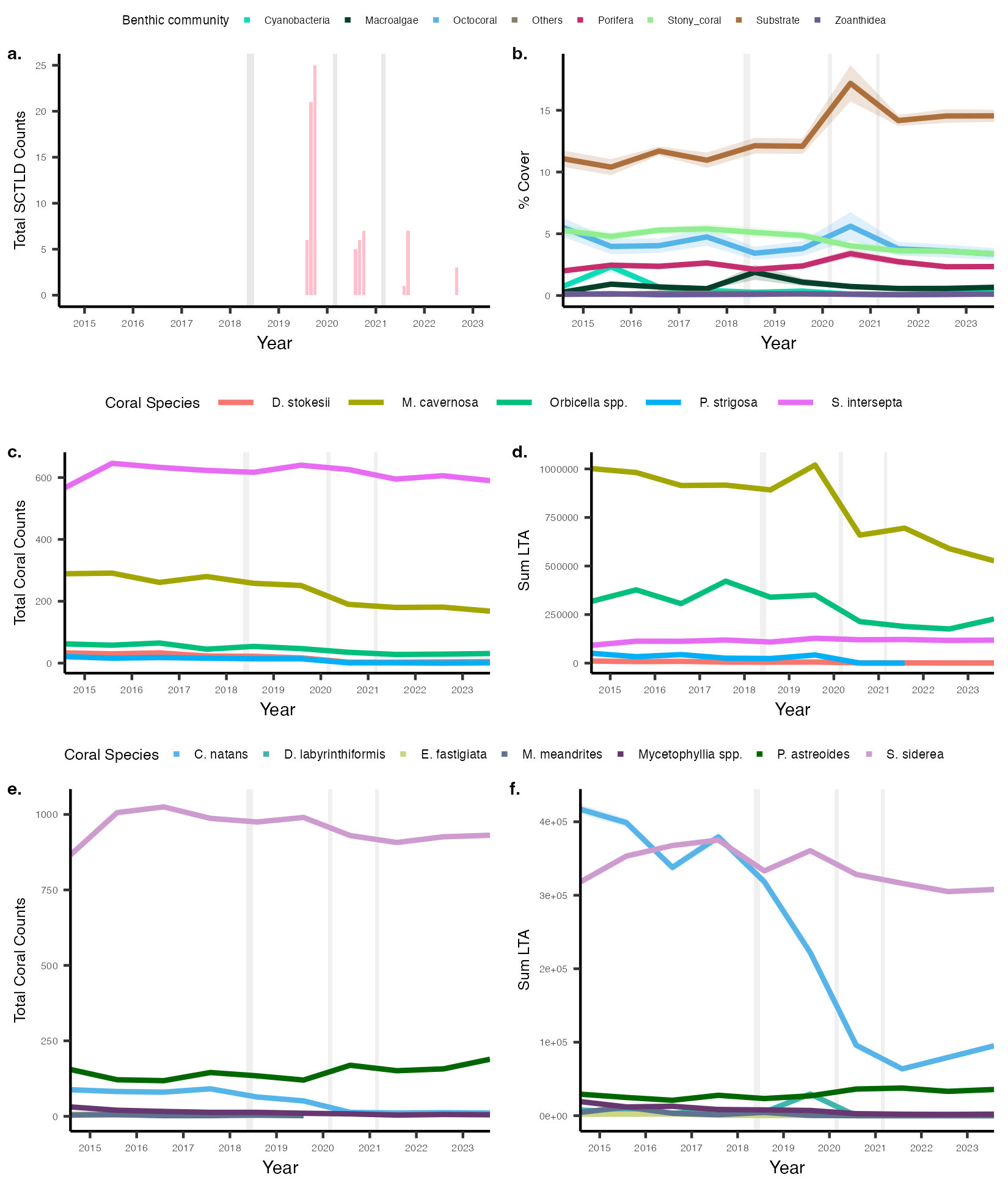

Supplement: Supplementary file 2 — Figure S1: Temporal trends in stony coral tissue loss disease (SCTLD) prevalence, benthic cover, coral counts, and live tissue area from 2014 to 2023 at Lower Keys patch reefs. (a) Monthly counts of SCTLD‐affected coral colonies at random sites from the Disturbance Response Monitoring (DRM) program. From four repeated‐measures sites in the Coral Reef Evaluation and Monitoring Project (CREMP), surveyed in the summer: (b) Percent cover of benthic groups, including macroalgae, cyanobacteria, stony corals, bare substrate and others, shown as mean annual percent cover ± SE for each group. (c) Total colony counts and (d) total living tissue area (LTA, cm2) for the five focal SCTLD‐susceptible coral species examined in this study (Dichocoenia stokesii, Montastraea cavernosa , Orbicella spp., Pseudodiploria strigosa and Stephanocoenia intersepta ), aggregated annually across CREMP sites. (d) Total colony counts and (f) LTA for seven other SCTLD‐susceptible or common coral species: Colpophyllia natans , Diploria labyrinthiformis , Eusmilia fastigiata , Meandrina meandrites , Mycetophyllia spp., Porites astreoides and Siderastrea siderea . Shaded grey bars in all panels denote microbiome sampling time: the vulnerable stage (May–June 2018), epidemic stage (February 2020) and endemic stage (February 2021). [file EMI4-17-e70264-s004.png]

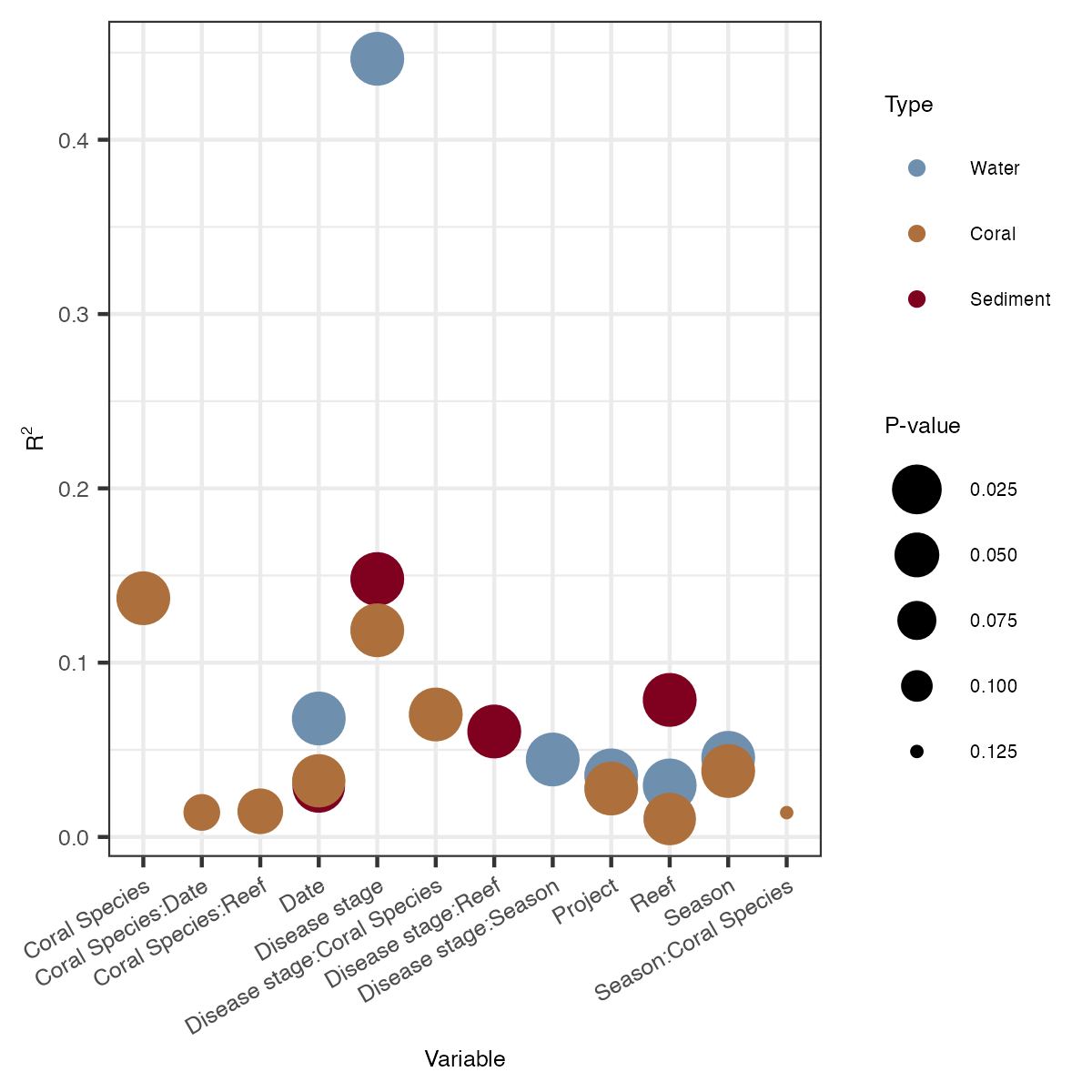

Supplement: Supplementary file 3 — Figure S2: Factors and their interactions were tested for explanatory value in structuring microbial communities. Shown are the R 2 values of each factor or interaction, with bubbles scaled to p values (the bigger the bubble, the smaller the p value), tested with PERMANOVA. ‘Coral species’ were Dichocoenia stokesii (DSTO), Stephanocoenia intersepta (SINT), Montastraea cavernosa (MCAV), Pseudodiploria strigosa (PSTR) and Orbicella faveolata (OFAV); ‘Date’ is sampling date (Figure 1); ‘Season’ represents wet or dry season (Table S1); ‘Reef’ represents three Lower Keys patch reefs (Figure 1); ‘Project’ refers to whether the samples came from Rosales et al. (2020), Clark et al. (2021) or new collections for this study; and ‘Disease stage’ is vulnerable, epidemic or endemic. [file EMI4-17-e70264-s006.png]

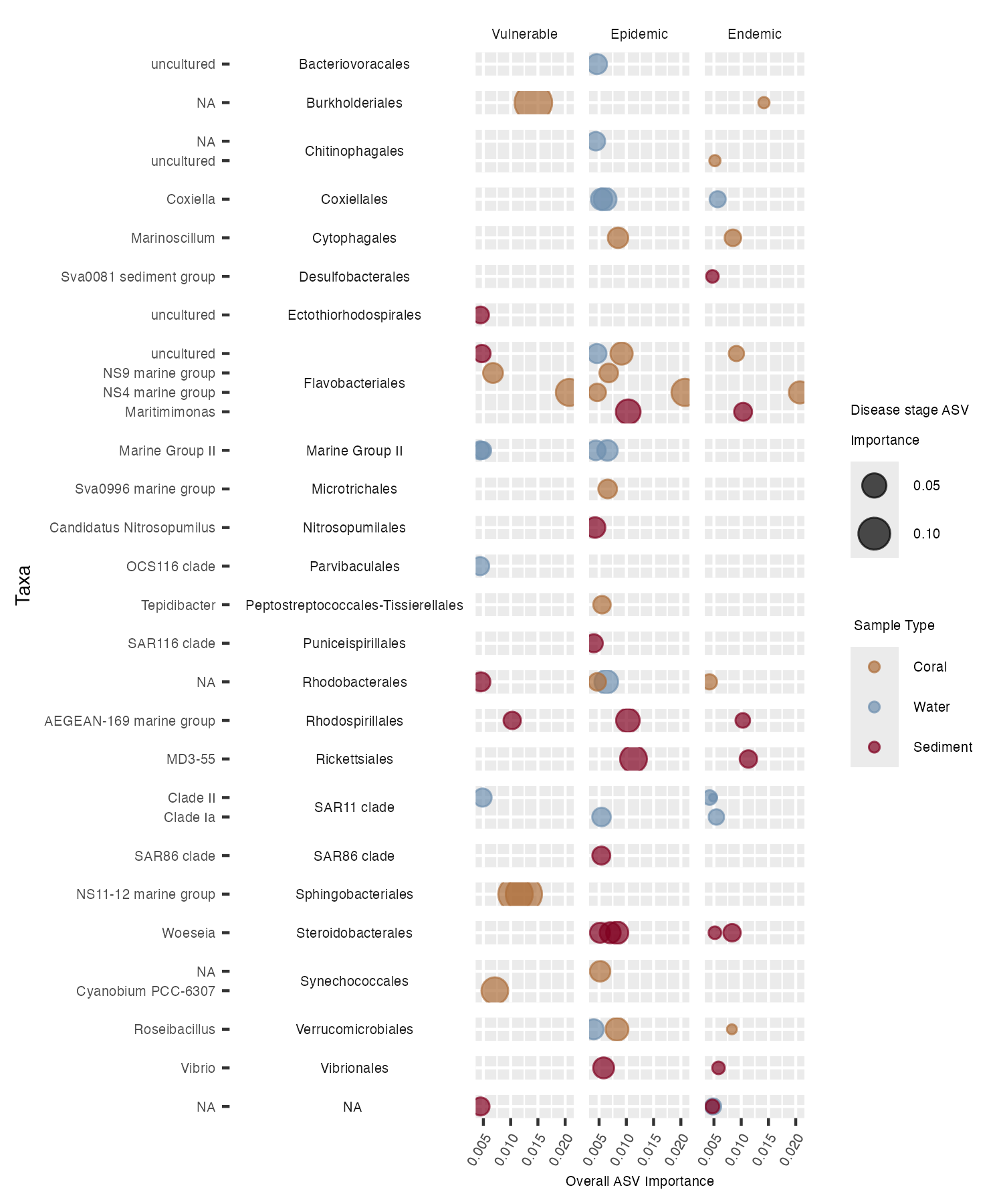

Supplement: Supplementary file 4 — Figure S3: The top 10 bacteria taxa per SCTLD stage and sample type, with the highest importance values assigned by random forest analysis. Bacteria amplicon sequence variants (ASVs) are coloured based on whether they were assigned as important in water, coral or sediment random forest analysis. The overall ASV importance is displayed on the x‐axis and grouped by disease stage. The y‐axis is grouped by bacteria order and then by genus. The bubble size indicates the importance of the ASV for each disease stage (i.e., vulnerable, epidemic or endemic). [file EMI4-17-e70264-s001.png]

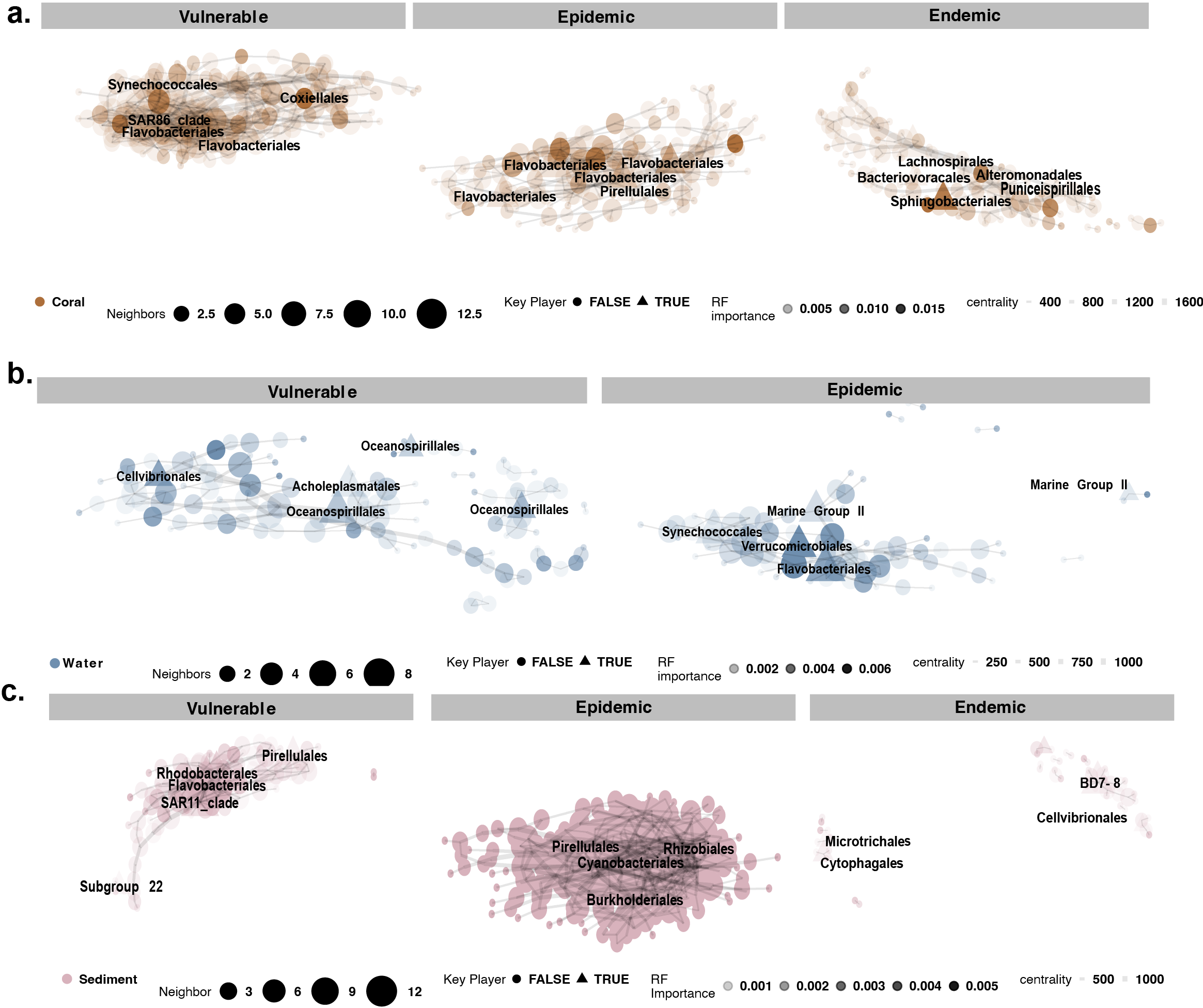

Supplement: Supplementary file 5 — Figure S4: Network analysis across SCTLD disease stages for (a) coral, (b) water, and (c) sediment. Nodes represent amplicon sequence variants (ASVs), with their size indicating the number of neighbouring ASVs. A triangle and label identifying the bacterial order signify that a node is a key player in the network. The width of the edges (i.e., the lines connecting the nodes) reflects centrality, with thicker edges indicating higher centrality. The alpha transparency of each point represents the overall importance of the random forest model. [file EMI4-17-e70264-s003.png]

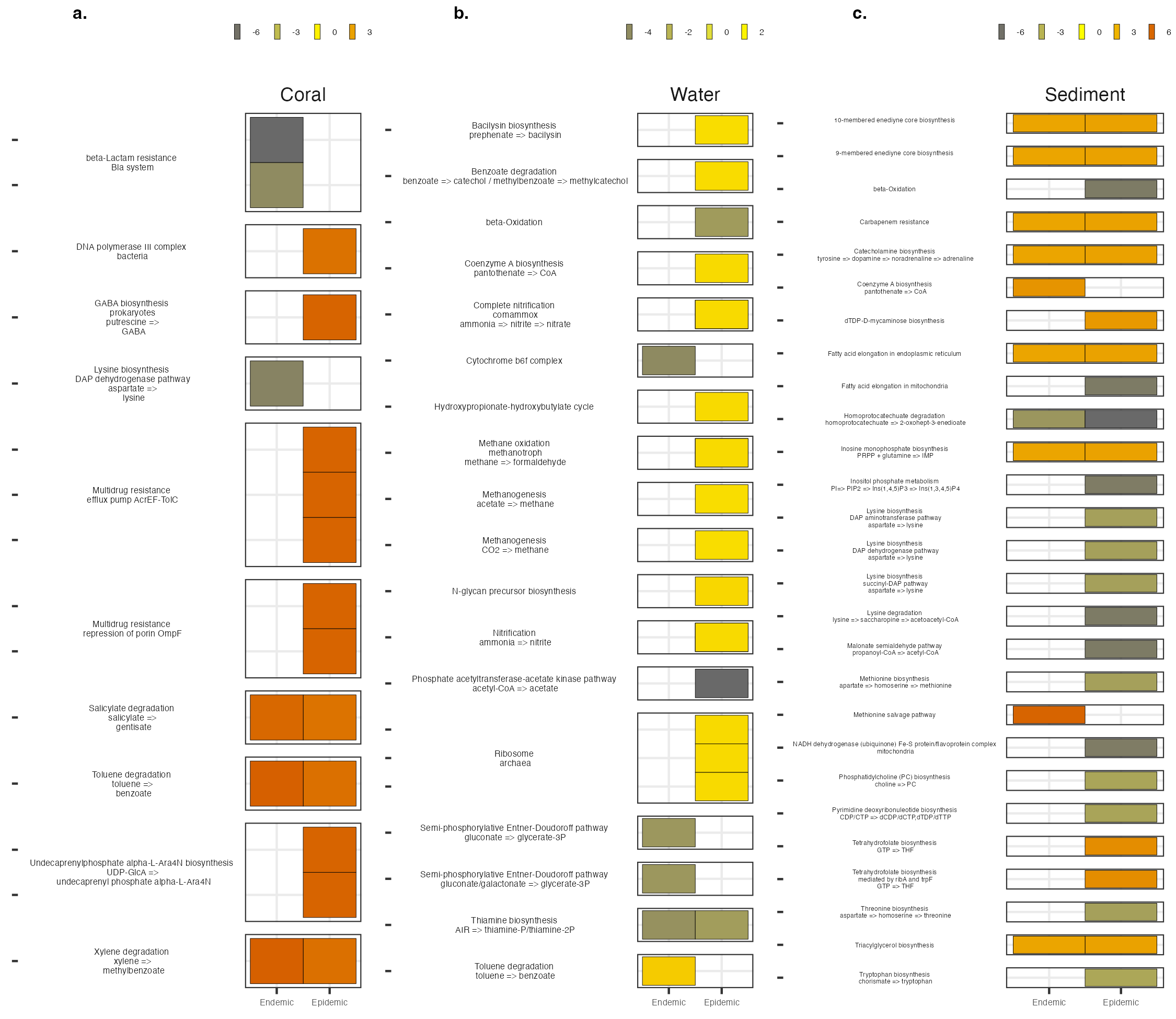

Supplement: Supplementary file 6 — Figure S5: Heatmaps show the predicted functions of the microbiome community for KEGG pathways of (a) coral, (b) water, and (c) sediments. The columns represent the disease stages, endemic and epidemic, relative to the vulnerable stage. The rows indicate different KEGG orthologies (KOs) and are grouped by modules. A negative coefficient or log fold change signifies enrichment in the vulnerable stage. [file EMI4-17-e70264-s007.png]
